# Pasteurized Bacteroides thetaiotaomicron and its extracellular vesicles improve metabolic profiles, expression of genes associated with diabetes and inflammation, and gut microbiota in type 2 diabetic rats

**DOI:** 10.17179/excli2025-8860

**Published:** 2025-12-04

**Authors:** Farzaneh Hasanian-Langroudi, Mehdi Hedayati, Asghar Ghasemi, Seyed Davar Siadat, Maryam Tohidi

**Affiliations:** 1 Prevention of Metabolic Disorders Research Center, Research Institute for Metabolic and Obesity Disorders, Research Institute for Endocrine Sciences, Shahid Beheshti University of Medical Sciences, Tehran, Iran; 2 Cellular and Molecular Endocrine Research Center, Research Institute for Endocrine Molecular Biology, Research Institute for Endocrine Sciences, Shahid Beheshti University of Medical Sciences, Tehran, Iran; 3 Endocrine Physiology Research Center, Research Institute for Endocrine Molecular Biology, Research Institute for Endocrine Sciences, Shahid Beheshti University of Medical Sciences, Tehran, Iran; 4 Department of Mycobacteriology and Pulmonary Research, Pasteur Institute of Iran, Tehran, Iran; 5 Microbiology Research Center, Pasteur Institute of Iran, Tehran, Iran

**Keywords:** Bacteroides thetaiotaomicron, extracellular vesicles, pasteurization, type 2 diabetes mellitus, gut microbiota

## Abstract

This study investigates the effect of pasteurized *Bacteroides thetaiotaomicron* (P*B.t*) and its extracellular vesicles (*B.t*-EVs) on metabolic parameters, diabetes- and inflammation-related gene expression, and microbiota composition in type 2 diabetes mellitus (T2DM). A total of forty-eight male Wistar rats were randomly divided into normal controls (NC, n=24) and T2DM-induced rats (n=24), and each group was further subdivided to receive phosphate-buffered saline (PBS), P*B.t*, or *B.t*-EVs by gavage daily for five consecutive weeks. The effects on obesity indices, glycemic markers, lipid profile, expression of diabetes- and inflammation-related genes in the liver and colon, and targeted changes in gut microbiota were assessed. Treatment with *B.t*-EVs and P*B.t *was associated with reductions in obesity indices (body weight, body mass index, and Lee index) and fasting blood glucose compared to the T2DM-PBS group; however, this reduction was significant only in T2DM-*B.t*-EVs rats (P≤0.0142). Both interventions yielded significant improvements in metabolic parameters, as demonstrated by decreased serum insulin, triglyceride, and total cholesterol levels, reduced homeostatic model assessment for insulin resistance (HOMA-IR), and improved glucose tolerance (all P≤0.0382). Both treatments reduced with downregulation of endocannabinoid system receptor 1 (CB1) expression and increased CB2 and phosphoinositide 3-kinase/protein kinase B (PI3K/Akt) gene expression in the liver (all P≤0.0018). In the colon, PB.t and B.t-EVs significantly downregulated interleukin (IL)-1β, IL-6, and CB1 genes. They also upregulated IL-4, IL-10, and CB2 genes (all P≤0.0004). Targeted microbiota analysis showed increased abundances of *Bacteroidetes*, *Faecalibacterium prausnitzii*, and *B.t*, accompanied by a reduced level of *Firmicutes*, *Actinobacteria* and *Firmicutes/Bacteroidetes* (*F/B*) ratio (P≤0.0492). Additionally, treatment with *B.t*-EVs increased the abundance of *Clostridium cluster* IV (P=0.0085). Histological findings indicated reduced pancreatic damage in the treated groups. Altogether, these results suggest that P*B.t *and *B.t*-EVs, as paraprobiotic and postbiotic candidates, may improve metabolic health, reduce inflammation, and modulate gut microbiota composition in T2DM.

See also the graphical abstract(Fig. 1).

## Introduction

Type 2 diabetes mellitus (T2DM), defined by hyperglycemia due to insulin resistance (IR), is a rising global health crisis that greatly elevates the risk of early mortality, with projections estimating 783 million cases by 2045 (Zhang et al., 2024[60]). Lifestyle choices, excess adiposity, and microbial imbalances (dysbiosis) exacerbate T2DM and intensify its socioeconomic impact. Faced with the side effects associated with standard diabetes therapies, targeting the gut microbiota offers a promising, safer, and innovative strategy to improve disease management and reduce treatment-related complications (Yu et al., 2025[58]). The endocannabinoid system (ECS), via its cannabinoid receptors 1 (CB1) and CB2, links gut microbiota to T2DM and offers new treatment targets (Chong et al., 2025[8]). Probiotics alter gut bacteria composition, attenuate inflammation, and enhance immune function, partly through ECS modulation. Activation of CB1 and CB2 influences the phosphoinositide 3-kinase/protein kinase B (PI3K/Akt) pathway. This improves glucose uptake, enhances insulin sensitivity, and suppresses inflammation. The cascade supports metabolic homeostasis and reduces pro-inflammatory cytokines such as interleukin-6 (IL-6) (Gioacchini et al., 2017[15], Lima et al., 2022[27]).

In addition to direct host-microbe interactions, microbial metabolites (collectively termed postbiotics) play a pivotal role in modulating host physiological functions. Postbiotics encompass components such as extracellular vesicles (EVs) and paraprobiotics, which are non-viable bacterial cells. These agents have attracted growing interest due to several advantages over traditional probiotics, including defined molecular composition, improved physicochemical stability, and greater suitability for industrial processing and storage. Furthermore, paraprobiotics may engage more selectively with host receptors, allowing for the precise activation of downstream biological pathways (Nataraj et al., 2020[32]). In parallel, EVs (20-250 nm) derived from gut Gram-negative bacteria carry proteins, enzymes, lipopolysaccharide (LPS), and nucleic acids, allowing them to interact with immune cells (Schwechheimer and Kuehn, 2015[39]). Numerous studies have documented the beneficial impact of EVs on the treatment of various disorders (Borrelli et al., 2018[5], Shi et al., 2023[40]). Postbiotic and paraprobiotic therapy of the gut microbiota is considered a viable approach for managing T2DM (Kim et al., 2024[21], Qu et al., 2025[36]).

Among next-generation probiotics (NGPs) as emerging microbial therapeutics, *Bacteroides spp.* is one of the most dominant genera in the healthy human intestine and is widely used due to its beneficial role in treating various diseases (Tan et al., 2019[45]). Notably, *Bacteroides thetaiotaomicron* (*B.t*), a representative of the phylum *Bacteroidetes* and a potential NGP, is a commensal Gram-negative gut bacterium. Importantly, *B.t'*s extracellular vesicles (*B.t*-EVs) reduce pro-inflammatory factors and boost anti-inflammatory mediators (Fonseca et al., 2022[12]). Furthermore, both pasteurized *B.t* (P*B.t*) and *B.t*-EVs regulate glucose homeostasis through gut hormone modulation, making them promising candidates for metabolic health interventions (Vaezijoze et al., 2025[51]). Therefore, they can be promising candidate for managing metabolic disorders, particularly T2DM.

Despite encouraging evidence, most previous studies have mainly provided findings on the overall metabolic effects of paraprobiotics and postbiotics derived from *B.t *(Lee et al., 2024[25], Vaezijoze et al., 2025[51]), but the relationship of these substances with metabolic regulation requires further investigation by focusing on insights into signaling and microbiota changes associated with T2DM. Therefore, we aimed to investigate the effects of P*B.t *and *B.t*-EVs in a high-fat diet/streptozotocin (HFD/STZ)-induced T2DM model in Wistar rats by measuring obesity indices, glycemic indices, lipid profile, expression of genes related to diabetes and inflammation, as well as the abundance of targeted fecal bacteria.

## Materials and Methods

### Preparation of B.t

*B.t* CCUG 10774 was acquired from the DSMZ (German collection of microorganisms and cell cultures) Institute, and cultured on Brain Heart Infusion (BHI) agar (Merck, Darmstadt, Germany) supplemented with 5 μg/mL hemin and 1 μg/mL menadione (Sigma-Aldrich, USA). Cultures were incubated anaerobically at 37 °C (80 % N₂, 10 % CO₂, 10 % H₂) using an Anoxomat™ MARK II system until reaching (optical density) OD 600 nm (OD_600_) of 1, following established protocols (Tafti et al., 2019[44]). 

### Preparation B.t-EVs

*B.t*-EVs were isolated following an anaerobic overnight incubation, as previously mentioned (Stentz et al., 2015[42]). To minimize contamination from membrane debris and cytosolic proteins released during late stationary-phase lysis, *B.t*-EVs were collected during the early stationary phase from BHI cultures, capturing vesicles produced during exponential growth. This approach prioritizes intact *B.t*-EVs reflecting the bacteria's active physiological state over degradation products formed during prolonged culture. Briefly, for isolation, *B.t* cultures were centrifuged (11,000 ×g, 20 min, 4 °C); the supernatant was vacuum-filtered (0.22 μm, SMC220; Biosorfa, China) to remove residual cells/debris, concentrated to 200 mL via ultrafiltration (pellicon® 88 cm^2^ & 0.11 M2 cassette hold, cat. #xx42pmini, Merck, Germany), and subjected to dual ultracentrifugation (Beckman, USA): first at 130,000 ×g (2 h, 4 °C), with pellets resuspended in 20 mL phosphate buffered saline (PBS), then at 200,000 ×g (2 h, 4 °C). Final pellets were resuspended in 1 mL PBS, filtered (0.22 μm), and stored at −80 °C. 

### Evaluation of B.t-EVs protein content 

**Figure 2:**
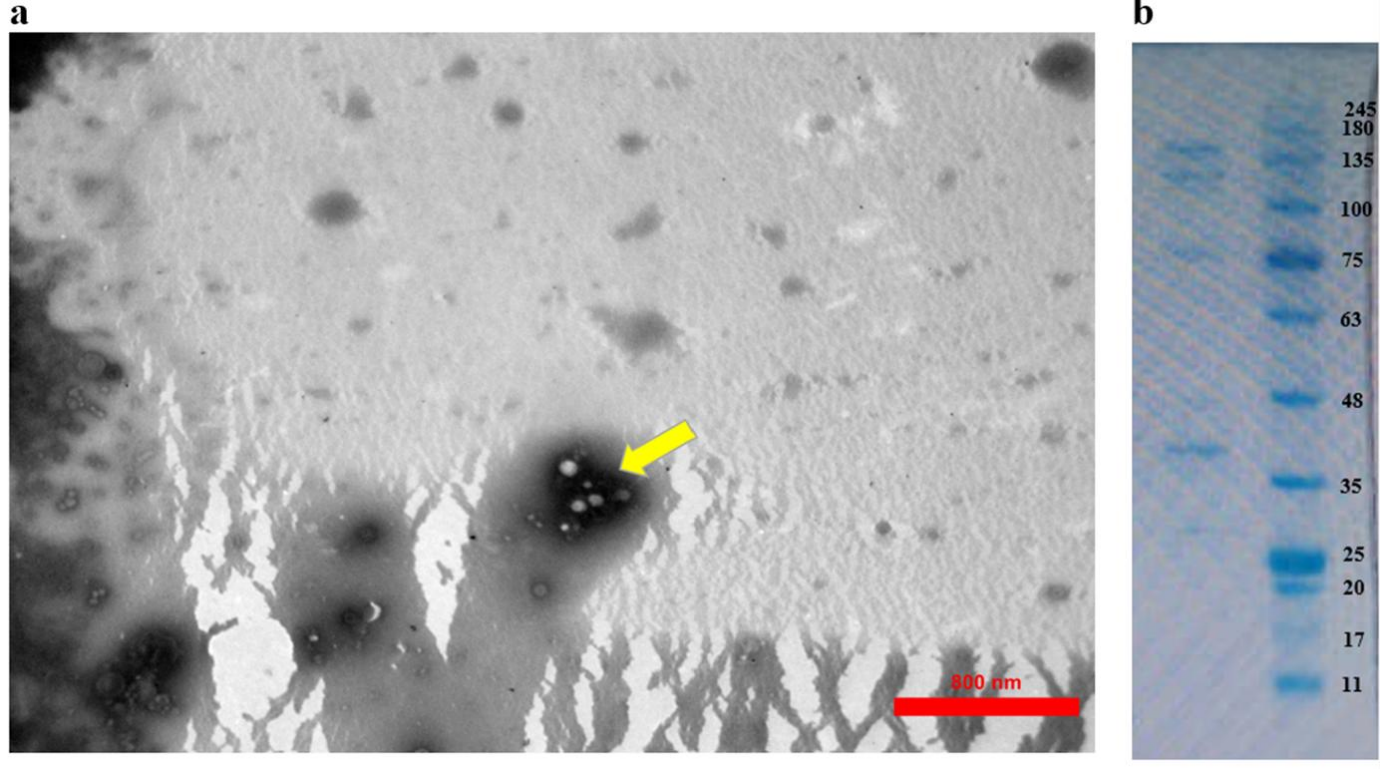
(a) Transmission electron microscopy: Spherical morphology of extracellular vesicles (scale bars, 800 nm), (b) The protein profile derived from *Bacteroides thetaiotaomicron* in sodium dodecyl sulfate polyacrylamide gel electrophoresis

Transmission electron microscopy (TEM) confirmed the morphology of EVs, revealing spherical particles sized between 30 and 110 nm, as previously described (Jones et al., 2020[20]) (Figure 2a(Fig. 2)).

The total protein concentration in purified *B.t*-EVs was first measured using a NanoDrop™ spectrophotometer (Thermo Scientific, Wilmington, DE, USA), followed by confirmation with the Bradford assay through absorbance measurement at 590 nm. The protein profile was then analyzed via 12 % sodium dodecyl sulfate polyacrylamide gel electrophoresis (SDS-PAGE) (Badi et al., 2019[3]), revealing protein bands ranging from 11 to 245 kDa, which indicated the protein composition of the EVs (Figure 2b(Fig. 2)). 

### Preparation of PB.t 

Fresh *B.t* was collected at an OD 600 nm (OD_600_) of 1 to prepare P*B.t *bacteria. The bacterial pellets were washed with sterile anaerobic PBS and adjusted to 1 × 10^9^ colony-forming units (CFU)/mL for daily oral administration to rats. The bacterial solution was then heat-inactivated at 70 °C for 30 minutes (Vaezijoze et al., 2025[51], Yinhe et al., 2024[57]). 

### Study design

In this study, a diabetic model was induced using STZ, an alkylating agent that leads to cell death, in combination with HFD. This model more accurately replicates the pathophysiological characteristics of human T2DM (Gheibi et al., 2017[14]). Previous research shows that female animals are less sensitive to the diabetogenic effect of STZ because estrogen interferes (Goyal et al., 2016[17]). Therefore, the current study enrolled 48 male Wistar rats (8 weeks old, weighing 190-210 g) obtained from the Pasteur Institute of Iran. Animals were kept in a controlled environment (22 ± 2°C, 40 ± 6 % humidity) under a 12/12-hour light-dark cycle (lights on at 06:00). Every 2-3 rats were housed in hygienic cages with autoclaved hardwood chip bedding and had unrestricted access to standard chow and autoclaved water. Animal handling was conducted in accordance with Iranian laboratory animal care guidelines (Ahmadi-Noorbakhsh et al., 2021[1]). Animal operations were approved by the Shahid Beheshti University of Medical Sciences ethics committee (IR.SBMU.ENDOCRINE.REC.1402.043).

After one week of acclimation, rats were randomly assigned to two groups. The normal control group (NC, n=16) received a standard chow as a normal diet (ND) (10 % kcal from fat; Khorak Dam Pars Co., Tehran, Iran), while the HFD group (n=16) was fed a diet containing 58 % fat, 27.5 % carbohydrates, and 14.5 % protein. The composition of the HFD is detailed in Supplementary Table 1. Following 3 weeks of dietary intervention, rats were fasted for 12 hours. Then, HFD-fed rats received an intraperitoneal injection of 30 mg/kg STZ (Sigma, St. Louis, MO, USA) in 0.1 M citrate buffer (pH 4.5); NC rats were injected with citrate buffer alone. One week later, rats with fasting blood glucose (FBG) levels between 150-350 mg/dL were considered to have T2DM (Gheibi et al., 2017[14]). 

**Figure 3:**
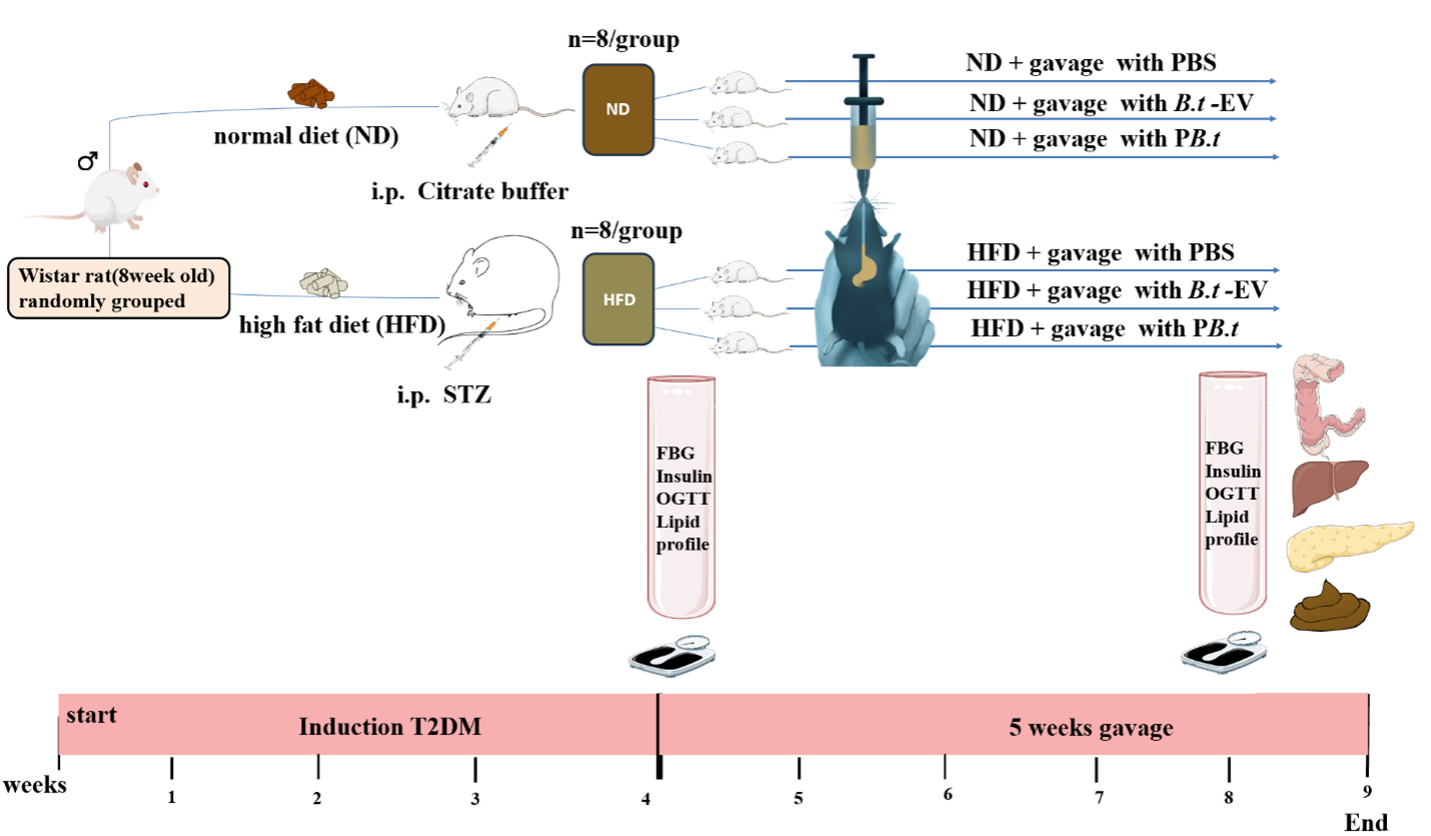
Study design of the animal experiment

Considering the FBG concentration, of 40 rats, 16 were excluded from the study [FBG <150 mg/dL (n = 10), FBG >350 mg/dL (n = 6)]. The remaining 24 rats (60 %) were classified as having T2DM. The T2DM rats were randomly divided into 3 subgroups (n=8 per group): a) T2DM-PBS (HFD + PBS); b) T2DM-*B.t*-EVs (HFD + *B.t*-EVs [50 µg protein in 1 mL PBS]) (Vaezijoze et al., 2025[51]); and c) T2DM-P*B.t* (HFD + P*B.t *[1×10⁹ CFU/mL in PBS]) (Yinhe et al., 2024[57]). Similarly, NC rats were randomly assigned to 3 subgroups (n=8 per group): a) NC-PBS (ND + PBS); b) NC-*B.t*-EVs (ND + *B.t*-EVs); and c) NC-P*B.t *(ND + P*B.t*). All treatments were gavaged orally for a period of 5 weeks. A schematic overview of the animal model and treatments is shown in Figure 3(Fig. 3). A sample size of 8 rats per group was determined based on the following formula (1) (Liu et al., 2023[29], Rosner, 2006[37]), n: sample size per group; *Z*_1-α/2_:23b? Z value corresponding to the selected confidence level (α is usually 0.05): *Z*_1-β_: Z value corresponding to the statistical power (β is usually 0.2, power = 80 %): S₁²: variance of the first group: S₂²: variance of the second group: d: expected difference between the means of the two groups (effect size).


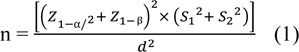


### Assessment of obesity indices

Body weight (BW), body mass index (BMI; BW [g]/length [cm²]), and Lee index (cube root of BW [g]/naso-anal length [cm]) were measured at week 4 (7 days post-STZ injection in T2DM rats and after buffer citrate injection in NC rats), and also at week 9 (after 5 weeks of intervention) (Ghasemi et al., 2021[13]).

### Laboratory assessment

Blood was collected from the tail tip of rats under sodium pentobarbital anesthesia (45 mg/kg) either after fasting or during the oral glucose tolerance test (OGTT), performed at both week 4 and week 9.

#### Biochemical markers assay in blood

Serum levels of FBG, total cholesterol (TC), triglycerides (TG), high-density lipoprotein-cholesterol (HDL-C), and low-density lipoprotein-cholesterol (LDL-C) were determined using commercial kits and serum controls (AUDIT, Delta Darman Part, Tehran, Iran), and a Pictus 700 chemistry analyzer (Diatron MI Plc, Budapest, Hungary). The intra- and inter-assay coefficients of variation for all biochemical tests were below 5.2 %. Serum fasting insulin levels were assessed using an enzyme-linked immunosorbent assay (ELISA) kit (Zellbio, Germany) with the Sunrise ELISA reader (Tecan Co., Salzburg, Austria). Homeostatic model assessment for IR (HOMA-IR) was calculated as (2):


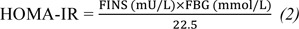


(Zeng et al., 2023[59]). 

#### Evaluation of glucose tolerance

For the OGTT, a glucose solution (2 g/kg of 50 % dextrose; Shahid Ghazi Pharmaceutical Co., Tabriz, Iran) was administered orally after a 12-hour fast. Blood samples were obtained at 0, 15, 30, 60, 90, and 120 minutes following administration (Kuo et al., 2021[23]).

### Tissue collection

Rats were anesthetized with intraperitoneal injections of xylazine (5 mg/kg) and ketamine (90 mg/kg) (Bremer Pharma GmbH, Germany) before being sacrificed (Tavares Pereira et al., 2012[48]). The pancreas, colon, and liver were excised, rinsed in cold PBS, and prepared for further analysis. Colon and liver tissues were snap-frozen in liquid nitrogen and stored at −80 °C for molecular studies, while pancreatic samples were fixed in 10 % neutral buffered formalin for tissue examination (Ningsih et al., 2021[33]).

#### Determination of target genes in liver and colon tissues 

Based on the KEGG database, genes related to T2DM in the liver and colon were selected for expression analysis. RNA was extracted from frozen liver and colon tissues using TRIzol reagent (Maxell, Iran) according to the manufacturer's protocol. Complementary DNA (cDNA) synthesized with a high-capacity reverse transcription kit (CinnaGene, Iran). Gene expression was quantified by Quantitative Polymerase Chain Reaction (qPCR) on a StepOne Real-Time qPCR System (Applied Biosystems, Life Technologies, Austin, TX, USA) using SYBR Green 2X Master Mix (Parstous, Iran). Liver samples were analyzed for CB1, CB2, PI3K, and Akt expression; colon samples for CB1, CB2, IL-6, IL-10, IL-4 and IL-1β. The elongation factor 2 (Eef2) served as the internal reference. Primer sequences are presented in Supplementary Table 2. Each qPCR run included positive and negative controls, and melt curve analysis confirmed specificity. Relative gene expression (fold change) was determined using the 2^-ΔΔCt^ method.

### Extraction of Fecal DNA and analysis of fecal microbiota 

Based on several database analyses (https://disbiome.ugent.be/, https://gmrepo.humangut.info/, https://microbiomedb.org/mbio/, and https://microbiomology.org/) and numerous studies investigating the gut microbiota in relation to T2DM have implicated four phyla as potential contributors to disease development, including *Firmicutes*, *Bacteroidetes*, *Actinobacteria*, *Proteobacteria* alongside 5 bacterial genera/ species, including *Akkermansia muciniphila* (*A. muciniphilia*), *Faecalibacterium prausnitzi (F. prausnitzi),* *Clostridium cluster IV, Lactobacillus spp*. and *B.t *were assessed (Cunningham et al., 2021[10], Gurung et al., 2020[18]). The copy number of each bacterium in the fecal samples was evaluated through qPCR. At the end of the study, fresh fecal samples were collected using sterile containers and immediately transferred into cryotubes to store at -80 °C. From each sample, 200 mg was weighed and homogenized in accordance with the kit manufacturer's protocol. Genomic DNA was isolated using the FavorPrep™ DNA Fecal Mini Kit (Favargen Biotech, Taiwan). DNA concentration and purity were evaluated with a Thermo Scientific NanoDrop™ spectrophotometer (U.S.A). Extracted DNA samples were stored at -80°C until further microbial analysis (Lu et al., 2019[30]). qPCR was performed to quantify bacterial composition by amplifying specific regions of the bacterial 16S rRNA gene using primers targeting different phyla and genera. The specificity of primers was validated through NCBI BLAST analysis. Primer details were presented in Supplementary Table 3. qPCR reactions were run in duplicate on a Rotor-Gene Q system (QIAGEN, Germany), with each 20 μL reaction containing 10 μL SYBR Green Master Mix, 1 μL DNA template, 0.5 μL of each primer (10 pmol/μL), and 8 μL nuclease-free water. Serial dilutions of DNA from standard *Escherichia coli *strains were used to generate a standard curve, enabling the quantification of bacterial DNA in fecal samples. The bacterial DNA copy number was normalized relative to genome sizes. Average cycle threshold (Ct) values were converted into copy numbers using a previously established formula (Yang et al., 2015[56]).

### Histological assay by hematoxylin and eosin (H&E) staining

The preserved pancreatic tissue in fixative solution (10 % neutral formalin) was processed through dehydration in graded alcohol, xylene transparency, and paraffin embedding. Then, the paraffin blocks were sectioned at a thickness of 5 μm and stained with H&E (Yaman et al., 2017[55]).

### Statistical analysis 

Differences among groups for obesity indices, biochemical markers, and OGTT results were evaluated using two-way analysis of variance (ANOVA). Areas under the curve (AUC) of blood glucose levels were calculated using the trapezoidal method to evaluate glucose tolerance. A one-way ANOVA was applied to compare gene expression related to diabetes and inflammation, as well as gut microbiota composition across the groups. One- and two-way ANOVA were followed by Bonferroni's post-hoc test. Results are presented as mean ± standard error of the mean (SEM), with statistical significance set at *P*<0.05. All data analyses were conducted using GraphPad Prism 8.0 (GraphPad Software Inc., CA, USA).

## Results

### Effect of PB.t and B.t-EVs on obesity indices

#### Intra-group comparison between weeks 4 and 9

Table 1(Tab. 1) and Supplementary Figure 1 (a, c, e) display obesity indices (BW, BMI, and Lee index) at weeks 4 and 9 in the different study groups. Compared to week 4, NC-PBS and NC-P*B.t* rats had higher BW (26 % and 18 %, respectively; *P*<0.0001) at week 9. Similarly, T2DM-PBS rats exhibited significantly higher BW (38 %), as well as higher values of BMI and Lee index (27 % and 7 %, respectively) (all *P*≤0.0184).

In both T2DM-P*B.t *and T2DM-*B.t*-EVs rats, significantly higher values of BW (24 % and 13 %, respectively; both *P*≤0.0104) were seen at week 9. Regarding the Lee index, a significantly lower value (6 %; *P*=0.0014) was observed only in T2DM-P*B.t *rats.

#### Inter-group comparison in weeks 4 and 9

As shown in Table 1(Tab. 1) and Supplementary Figure 1 (a, c, e), no significant differences in obesity indices were observed among the groups before the intervention or at week 4. By week 9, T2DM-PBS rats exhibited significantly higher BW (13 %), BMI (29 %), and Lee index (11 %) than NC-PBS rats (all *P*≤0.0024). At the end of the study, both T2DM-P*B.t *and T2DM-*B.t*-EVs rats showed lower values of BW, BMI, and Lee index than T2DM-PBS rats. The respective reductions in T2DM-P*B.t *rats were 10 %, 21 %, and 9 %, and in T2DM-*B.t*-EVs rats were 19 %, 26 %, and 11 % (all *P*≤0.0011).

#### Inter-group comparison over 5 weeks through AUC analysis

As presented in Supplementary Figure 1(b, d, f), T2DM-PBS rats showed significantly higher BW, BMI, and Lee index (8 %, 17 % and 6 %, respectively) than NC-PBS rats over the 5-week period (*P*≤0.0487). In contrast, among treated T2DM rats, *B.t*-EVs rats exhibited significantly lower BW, BMI, and Lee index (11 %, 17 % and 7 %, respectively) compared to T2DM-PBS rats (*P*≤0.0142(. Additionally, while treatment with P*B.t* was also associated with a reduction of obesity indices, these decreases were not statistically significant.

### Effect of PB.t and B.t-EVs on biochemical parameters

#### Intra-group comparison between weeks 4 and 9

The biochemical parameters (FBG, insulin, HOMA-IR, and lipid profile) at weeks 4 and 9 in different study groups are shown in Table 1(Tab. 1) and Supplementary Figure 2. Compared to week 4, T2DM-PBS rats had significantly higher TG (36 %), insulin (83 %), and HOMA-IR (89 %) (all *P*≤0.0051) at week 9. In T2DM-P*B.t *rats, FBG, TC, and LDL-C were significantly lower, with corresponding values of 8 %, 21 %, and 23 %, respectively (all *P*≤0.0067). Similarly, T2DM-*B.t*-EVs rats had significantly lower FBG (10 %), TC (17 %), LDL-C (18 %); furthermore, this group had 21 % higher HDL-C compared to week 4 (all *P*≤0.0346). [Table 1(Tab. 1), and Supplementary Figure 2 (a, c, e, g, i, k, m)].

#### Inter-group comparison in weeks 4 and 9

At both weeks 4 and 9, compared to NC-PBS rats, T2DM-PBS rats had significantly higher FBG (108 % and 105 %), insulin (569 % and 966 %), HOMA-IR (1283 % and 2115 %), TG (114 % and 131 %), TC (26 % and 32 %) and LDL-C (42 % and 50 %) (all *P*≤0.0019).

In comparison between treated rats and T2DM-PBS rats, no significant differences were seen at week 4. In T2DM-P*B.t *had significantly lower levels of FBG (15 %), insulin (40 %), HOMA-IR (53 %), TG (27 %), TC (24 %), and LDL-C (26 %) at week 9 (all *P*≤0.013). Similarly, T2DM-*B.t*-EVs rats demonstrated significantly lower FBG (18 %), insulin (55 %), HOMA-IR (63 %), TG (37 %), TC (25 %), and LDL-C (23 %), with higher HDL-C (38 %) (all *P*≤0.0087), compared to T2DM-PBS rats. [Table 1(Tab. 1), and Supplementary Figure 2 (a, c, e, g, i, k, m)].

#### Inter-group comparison over 5 weeks through AUC analysis 

As demonstrated in Supplementary Figure 2 (b, d, f, h, j, l, n) T2DM-PBS rats had significantly higher levels of FBG (107 %), insulin (781 %), HOMA-IR (1734 %), TG (123 %), TC (29 %), and LDL-C (46 %) compared to NC-PBS rats over 9 weeks (all *P*<0.0001), and a non-significant lower HDL-C (15 %) which, although not statistically significant, may still suggest a biologically favorable trend.

Compared to T2DM-PBS rats, both T2DM-P*B.t and *T2DM-*B.t*-EVs rats had significantly lower insulin, HOMA-IR, TG and TC over the 5-weeks period (31 %, 41 %, 15 %, and 12 % respectively for the former group; all *P*≤0.0335), the corresponding values were 43 %, 51 %, 25 %, and 15 % for the latter group (all *P*≤0.001). Although in both T2DM-P*B.t *and T2DM-*B.t*-EVs groups, FBG was decreased (10 % and 13 %, respectively), this decrement was just significant in T2DM-*B.t*-EVs rats *(P=*0.0114*)*. Additionally, T2DM-*B.t*-EVs rats exhibited a significantly higher HDL-C level (22 %; *P*=0.0313).

#### Effect of PB.t and B.t-EVs on OGTT 

**Figure 4:**
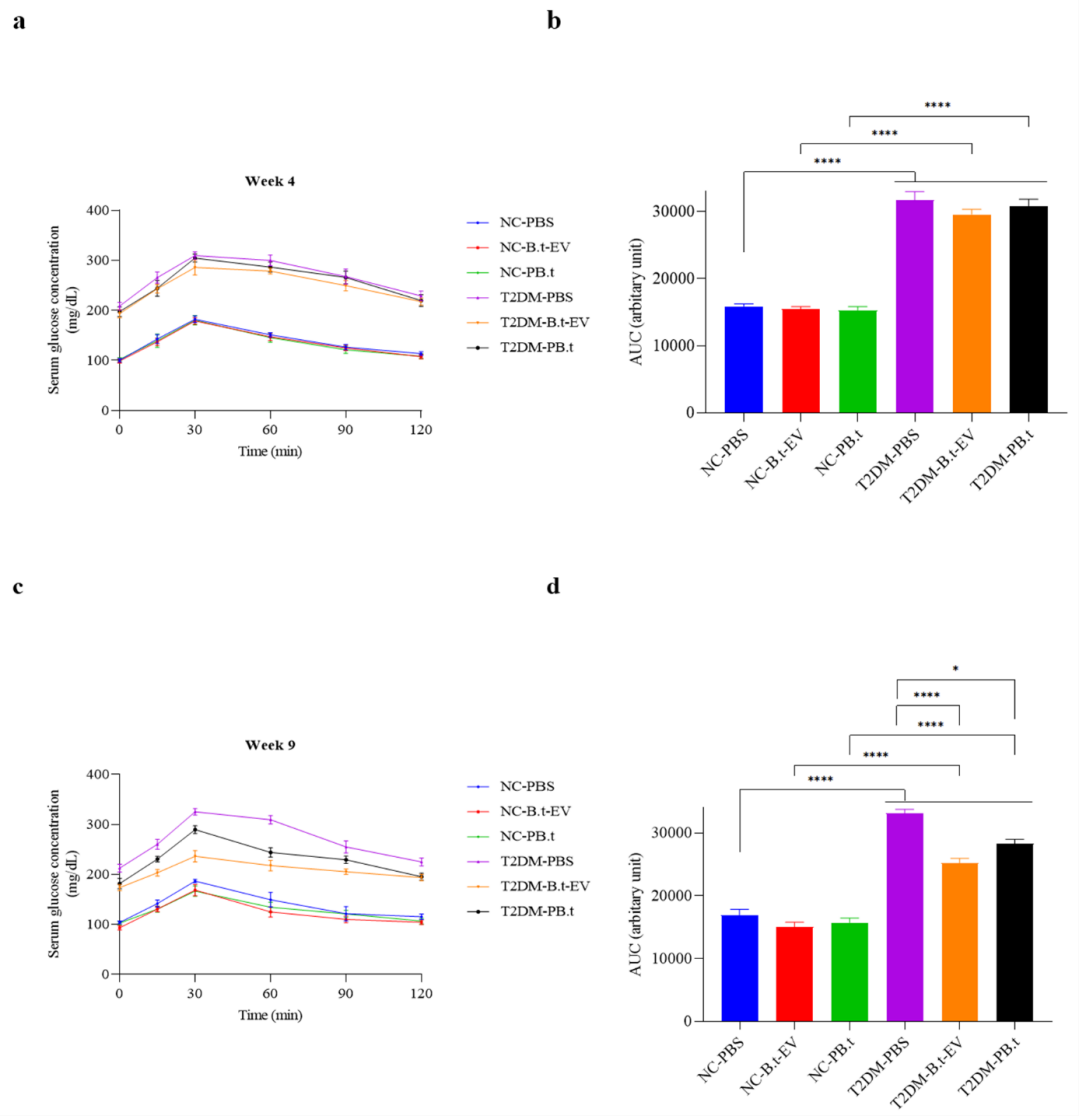
The effect of P*B.t *and *B.t*-EVs on OGTT (oral glucose tolerance test) at (a) week 4 and (b) AUC, (c) week 9 and (d) AUC, following intervention in rats. Data are expressed as mean ± SEM (standard error of mean) (n = 8/group); *, *P < *0.05; **, *P < *0.01; ***, *P < *0.001; ****, *P* < 0.0001 by post hoc Bonferroni's Two-way analysis of variance (ANOVA) *, statistically significant differences between groups in weeks 4 and 9. Groups categories: NC-PBS (normal control rats gavaged with PBS); NC-*B.t*-EV (NC gavaged with B*acteroides thetaiotaomicron* (*B.t)*'s Extracellular Vesicles); NC-P*B.t* (NC gavaged with Pasteurized *B.t*); T2DM-PBS (type 2 diabetes mellitus gavaged with PBS); T2DM-*B.t*-EV (T2DM rats gavaged with *B.t*-EV); T2DM-P*B.t* (T2DM rats gavaged with P*B.t)*.

In addition to the evaluation of FBG, we conducted OGTT to evaluate whether P*B.t *and *B.t-*EVs improved IR (Figure 4(Fig. 4)). In weeks 4 and 9, T2DM-PBS rats had glucose intolerance presented by significantly higher AUC (100 % and 85 %, respectively; *P*<0.0001) than NC-PBS rats. Compared to T2DM-PBS, significant reductions in AUCs were observed in week 9 in both T2DM-P*B.t *(10 %) and T2DM-*B.t*-EVs (19 %) groups (both *P≤*0.0382).

### Effects of PB.t and B.t-EVs on gene expression of the PI3K, Akt, CB1, and CB2 in the liver tissue of rats 

**Figure 5:**
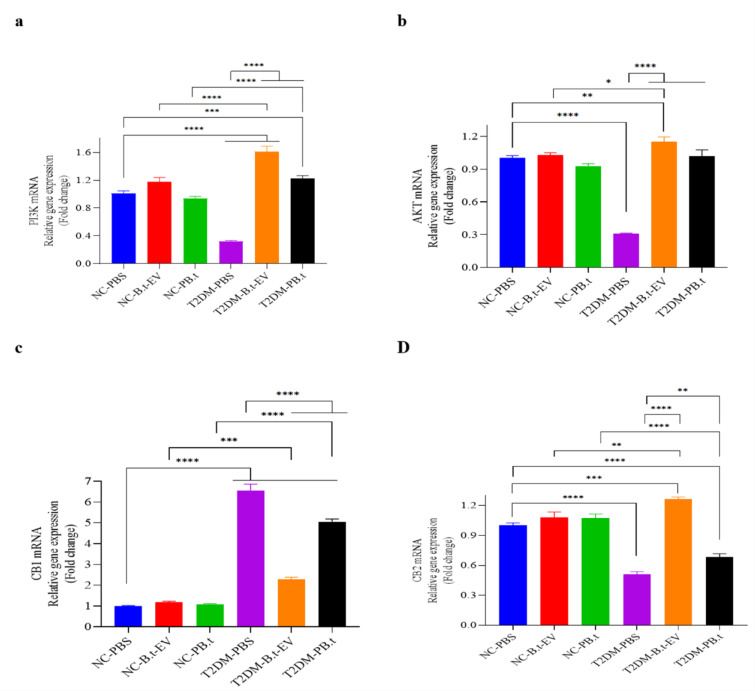
The effect of P*B.t *and *B.t*-EVs on the mRNA expression of liver diabetes-related genes; (a) PI3k (Phosphoinositide 3-kinase), (b) Akt (Protein Kinase B), (c) CB1 (Cannabinoid Receptor Type 1), and (d) CB2 (Cannabinoid Receptor Type 2). Data are expressed as mean ± SEM (standard error of mean) (n = 8/group); *, *P < *0.05; **, *P < *0.01; ***, *P < *0.001; ****, *P* < 0.0001 by post hoc Bonferroni's One-way analysis of variance (ANOVA). Groups categories: NC-PBS (normal control rats gavaged with PBS); NC-*B.t*-EV (NC gavaged with *Bacteroides thetaiotaomicron *(*B.t)*'s Extracellular Vesicles); NC-P*B.t* (NC gavaged with Pasteurized *B.t*); T2DM-PBS (type 2 diabetes mellitus gavaged with PBS); T2DM-*B.t*-EV (T2DM rats gavaged with *B.t*-EV); T2DM-P*B.t* (T2DM rats gavaged with P*B.t)*.

As illustrated in Figure 5(Fig. 5), gene expression analysis revealed that CB1 was significantly upregulated (6.54-fold), whereas PI3K (3.14-fold), Akt (3.24-fold), and CB2 (1.96-fold) were markedly downregulated in T2DM-PBS rats compared to NC-PBS rats (all *P*<0.0001). However, by both P*B.t* and *B.t*-EVs treatments the directions of changes were different i.e., downregulated expression of CB1 (1.29- and 2.88-fold, respectively) and upregulated expression of PI3K, Akt, and CB2 (3.80-, 3.29-, and 1.34-fold for T2DM-P*B.t *rats) and (5.01- 3.72-, and 2.47-fold, for T2DM-* B.t*-EVs rats) compared to T2DM-PBS (all *P*≤0.0018). 

### Effects of PB.t and B.t-EVs on gene expression of the IL-1β, IL-6, IL-4, IL-10, CB1, and CB2 in the colon tissue of rats 

**Figure 6:**
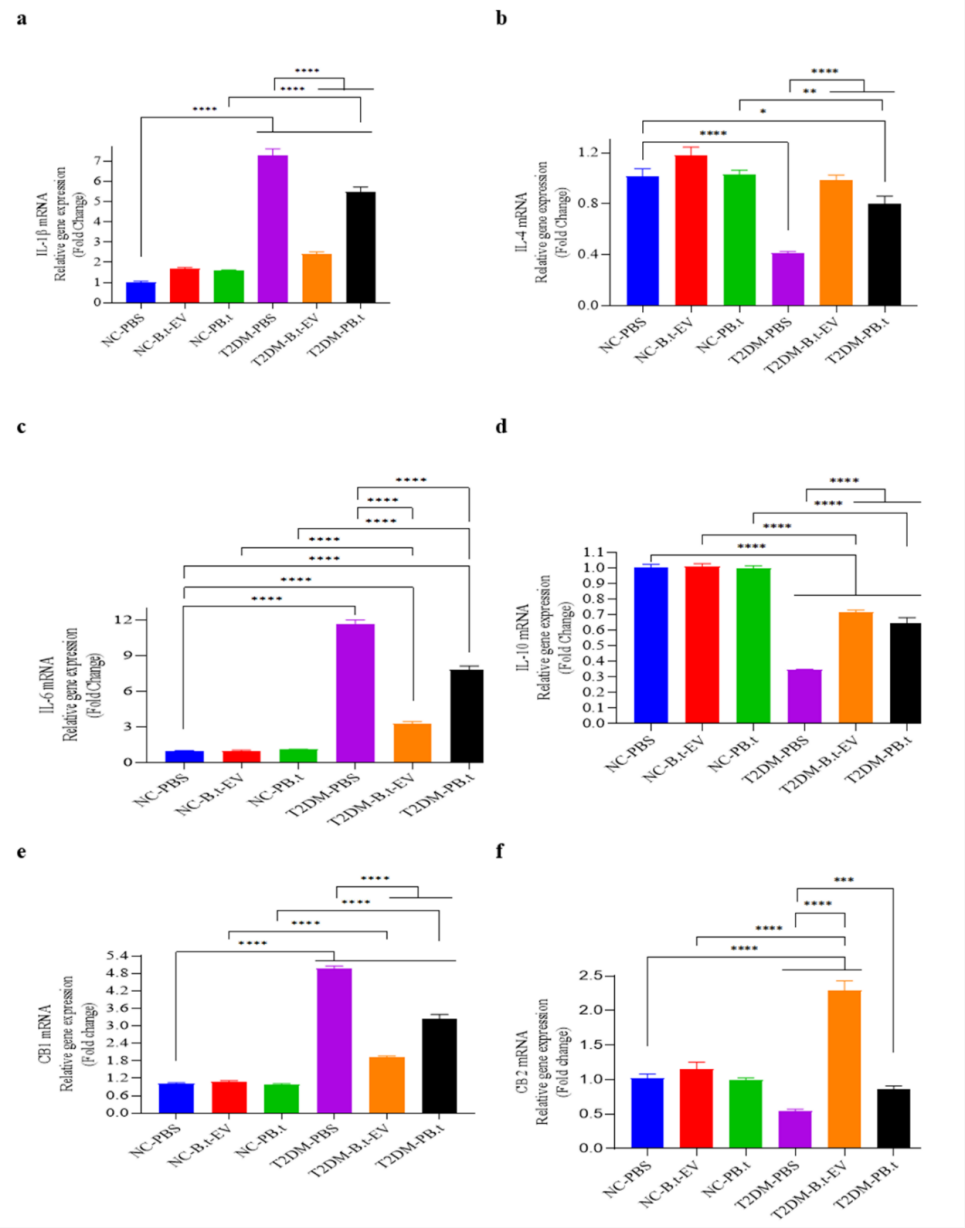
The effect of P*B.t *and *B.t*-EVs on the mRNA expression of inflammatory-related genes in rat colon tissue. (a) Il-1β (Interleukin-1 beta), (b) IL-4 (Interleukin-4), (c) IL-6 (Interleukin-6), (d) IL-10 (Interleukin-10), (e) CB1 (Cannabinoid Receptor Type 1), and (f) CB2 (Cannabinoid Receptor Type 2). Data are expressed as mean ± SEM (standard error of mean) (n = 8/group); *, *P < *0.05; **, *P < *0.01; ***, *P < *0.001; ****, *P* < 0.0001 by post hoc Bonferroni's One-way analysis of variance (ANOVA). Groups categories: NC-PBS (normal control rats gavaged with PBS); NC-*B.t*-EV (NC gavaged with *Bacteroides thetaiotaomicron* (*B.t)*'s Extracellular Vesicles); NC-P*B.t* (NC gavaged with Pasteurized *B.t*); T2DM-PBS (type 2 diabetes mellitus gavaged with PBS); T2DM-*B.t*-EV (T2DM rats gavaged with *B.t*-EV); T2DM-P*B.t* (T2DM rats gavaged with P*B.t)*.

As shown in Figure 6(Fig. 6), IL-1β, IL-6, and CB1 gene expression were significantly upregulated in T2DM-PBS rats (7.20-, 11.66-, and 4.92-fold, respectively), compared to NC-PBS rats (all *P*≤0.0001). In contrast, IL-4, IL-10, and CB2 expression markedly downregulated (2.47-, 2.89-, and 1.86-fold, respectively) (all *P*≤0.0088). After P*B.t *and *B.t-*EVs treatment, IL-1β, IL-6, and CB1 were significantly downregulated (1.33- 1.49-, 1.53-fold for T2DM- P*B.t *rats, and 3.06-, 3.57-, and 2.58-fold for T2DM-*B.t-*EVs rats), all *P*<0.0001. Furthermore, these treatments significantly upregulated IL-4, IL-10, and CB2 (1.95-, 1.86-, and 1.57-fold for T2DM- P*B.t *rats, and 2.40-, 2.07- and 4.20-fold for T2DM-*B.t-*EVs rats) compared to T2DM-PBS rats (all *P*≤0.0004). 

### Effect of PB.t and B.t-EVs on the targeted gut bacteria composition in T2DM rats 

**Figure 7:**
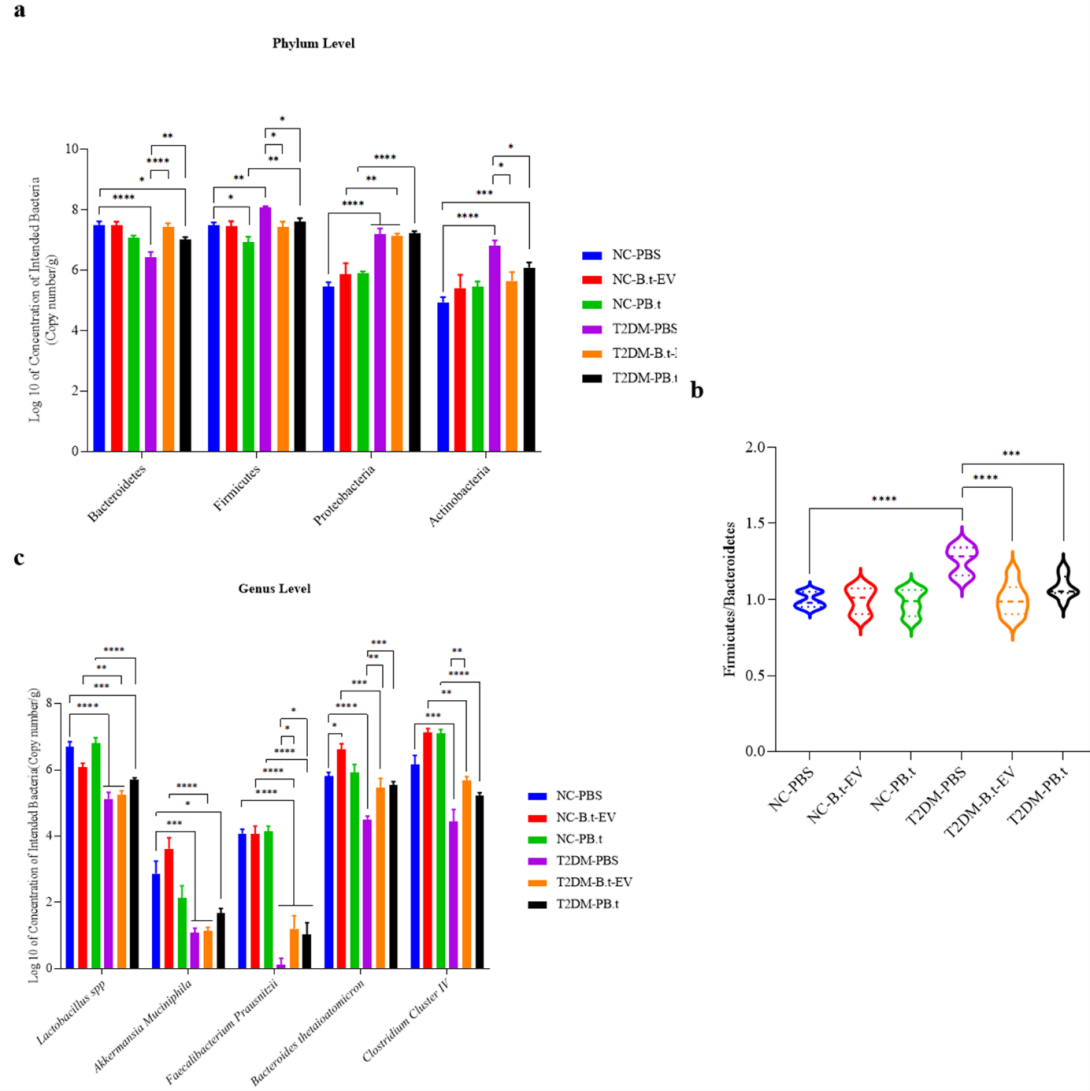
The effect of P*B.t *and *B.t*-EVs on the fecal targeted gut microbiota. (a) Gut microbiome composition at the phylum level, (b) Ratio of *Firmicutes* to *Bacteroidetes*, and (c) Gut microbiome composition at the genus/species level. Data are expressed as mean ± SEM (standard error of mean) (n = 8/group); *, *P < *0.05; **, *P < *0.01; ***, *P < *0.001; ****, *P* < 0.0001 by post hoc Bonferroni's One-way analysis of variance (ANOVA). Groups categories: NC-PBS (normal control rats gavaged with PBS); NC-*B.t*-EV (NC gavaged with *Bacteroides thetaiotaomicron (B.t)*'s Extracellular Vesicles); NC-P*B.t* (NC gavaged with Pasteurized *B.t*); T2DM-PBS (type 2 diabetes mellitus gavaged with PBS); T2DM-*B.t*-EV (T2DM rats gavaged with *B.t*-EV); T2DM-P*B.t* (T2DM rats gavaged with P*B.t)*.

**Figure 8:**
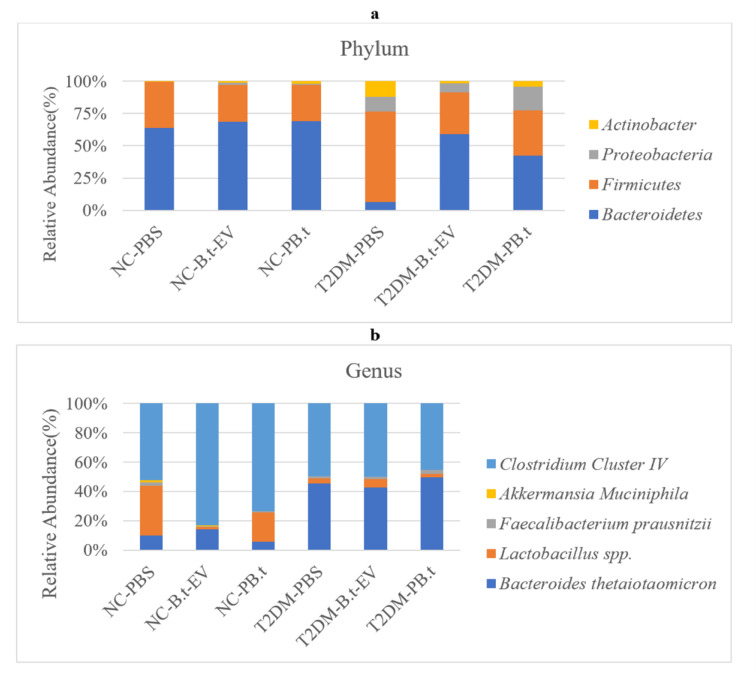
Column charts showing the population percentage of each bacterium in rats. (a) Relative abundance of bacteria at the phylum levels in NC-PBS, NC-*B.t*-EV, NC-P*B.t*, T2DM-PBS, T2DM-*B.t*-EV and T2DM-*B.t*. (b) Relative abundance of bacteria at the genus/species levels in NC-PBS, NC-*B.t*-EV, NC-P*B.t*, T2DM-PBS, T2DM-*B.t*-EV and T2DM-*B.t*. Groups categories: NC-PBS (normal control rats gavaged with PBS); NC-*B.t*-EV (NC gavaged with *Bacteroides thetaiotaomicron(B.t)*'s Extracellular Vesicles); NC-P*B.t* (NC gavaged with Pasteurized *B.t*); T2DM-PBS (type 2 diabetes mellitus gavaged with PBS); T2DM-*B.t*-EV (T2DM rats gavaged with *B.t*-EV); T2DM-P*B.t* (T2DM rats gavaged with P*B.t)*.

We examined copy numbers of targeted bacteria in fecal samples. According to Figures 7a(Fig. 7) and 8a(Fig. 8) at the phyla levels, our data demonstrated that compared to NC-PBS rats, copy numbers of *Firmicutes*, *Proteobacteria*,* and Actinobacteria *were significantly higher in T2DM-PBS rats (8 %, 32 %, and 38 %, respectively; all *P*≤0.0005) with their respective relative abundances of 71 %, 12 %, and 12 % versus 35 %, <1 %, and <1 % in NC-PBS rats. While the copy numbers of *Bacteroidetes *were lower (14 %; *P*<0.0001) with a relative abundance of 6 % versus 64 % in NC-PBS rats. 

After treatment, copy numbers of *Firmicutes *were lower in T2DM-P*B.t* and T2DM-*B.t*-EVs than in T2DM-PBS rats (6 % and 8 %, respectively; both *P≤*0.047), with the respective relative abundances of 35 % and 31 % versus 71 % in T2DM-PBS rats. Similarly, the copy numbers of *Actinobacteria *were also lower (11 % and 17 % for T2DM-P*B.t* and T2DM-*B.t*-EVs, respectively; both *P≤0.0463*) with their respective relative abundances of 4 % for T2DM-P*B.t* and 2 %for T2DM-*B.t*-EVs, versus 12 % in T2DM-PBS. However, copy numbers of* Bacteroidetes* were higher in both treated groups [15 % (relative abundance: 43 %) for T2DM-P*B.t* rats and 9 % (relative abundance: 60 %) for T2DM-*B.t*-EVs rats; both *P*≤0.0091] compared to T2DM-PBS rats (relative abundance: 6 %).

The *Firmicutes* to *Bacteroidetes* (*F/B*) ratio was significantly higher in the T2DM-PBS rats, as compared to the NC-PBS rats (26 %; *P*<0.0001). In addition, the *F/B* ratio in the T2DM-P*B.t *and T2DM-*B.t*-EVs groups was significantly lower (14 % and 20 %, respectively; both *P*≤0.0006) compared to the T2DM-PBS rats (Figure 7b(Fig. 7)). 

At the genus/species level, copy numbers of *Lactobacillus spp. *(24 %), *A. muciniphila *(63 %),* F. prausnitzii* (97 %)*, B.t (*23 %) and* Clostridium Cluster IV* (*28 %*) (all *P*≤0.0003) and their respective relative abundances 2 %, <1 %, 1 %, 46 % and 50 % were lower in T2DM-PBS compared to the NC-PBS rats (34 %, 2 %, 2 %, 10 %, and 52 %, respectively). We observed that, compared to T2DM-PBS, treatment with P*B.t *and *B.t*-EVs was significantly associated with higher copy numbers of *F. prausnitzii *(734 % and 873 %, respectively; both* P*<0.0492), with their respective relative abundances of 3 % in T2DM-P*B.t* and 2 % in T2DM-*B.t*-EVs, versus 1 % in T2DM-PBS. Similarly, copy numbers of* B.t* was also higher (23 % and 21 % in T2DM-P*B.t* and T2DM-* B.t*-EVs rats, respectively; both* P*<0.0001) and their respective relative abundances were 49 % for T2DM-P*B.t*, 43 % for T2DM-*B.t*-EVs, versus 46 % in T2DM-PBS, while a significantly higher copy number of *Clostridium Cluster IV* (28 %) with relative abundance of 49 % was only observed in T2DM-*B.t*-EVs (*P*=0.0085) (Figures 7c(Fig. 7) and 8b(Fig. 8)). 

### Effect of PB.t and B.t-EVs on histopathological changes of the pancreatic islets

**Figure 9:**
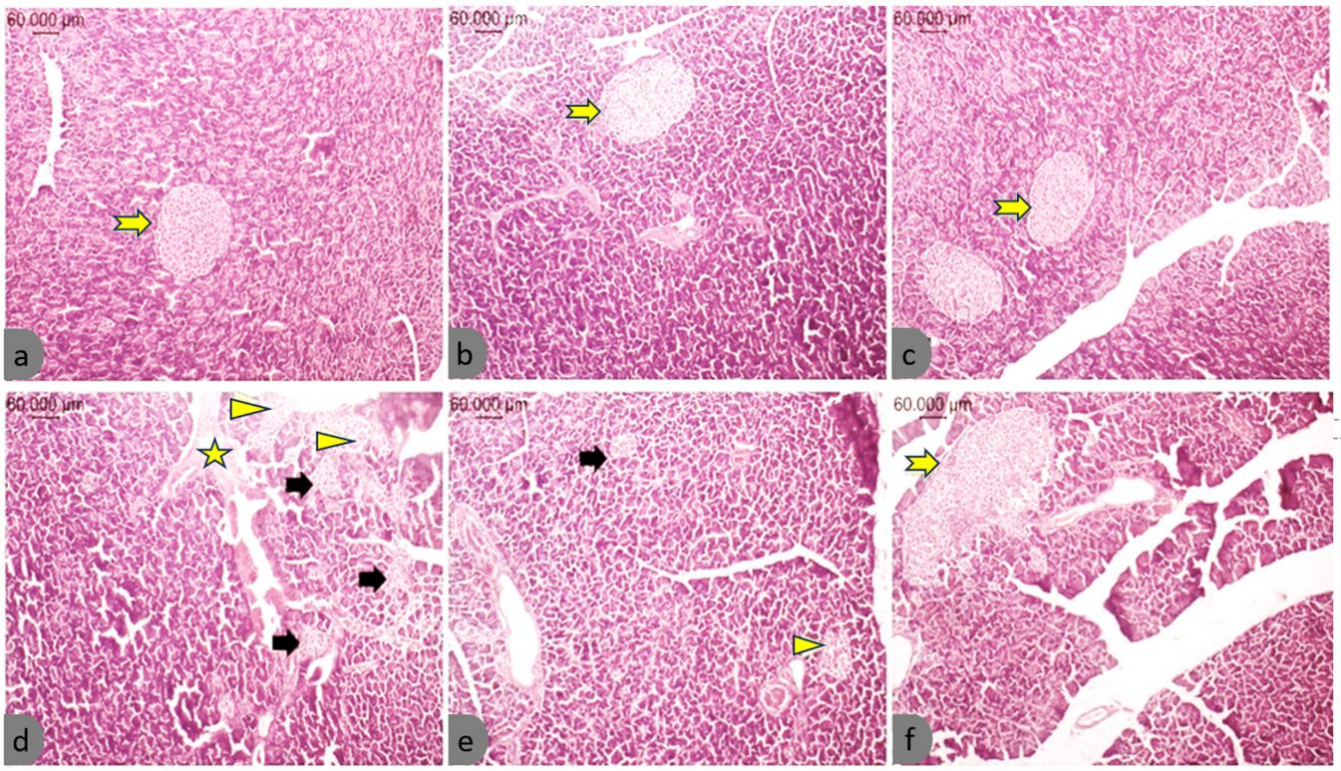
Histopathological findings of the pancreatic tissues in the different study groups. (a) NC-PBS, (b) NC-P*B.t*, (c) NC-*B.t*-EVs, (d) T2DM-PBS, (e) T2DM-P*B.t*, (f) T2DM-*B.t*-EVs; Pancreatic specimens were prepared and examined under a light microscope (Olympus SZX16, Japan) H&E stain;10X magnification. Normal islets (yellow arrow), islet atrophy (black arrow), destruction and vacuolization. (arrowhead), eosinophilic accumulations (star). Groups categories: NC-PBS (normal control rats gavaged with PBS); NC-*B.t*-EV (NC gavaged with *Bacteroides thetaiotaomicron(B.t)*'s Extracellular Vesicles); NC-P*B.t* (NC gavaged with Pasteurized *B.t*); T2DM-PBS (type 2 diabetes mellitus gavaged with PBS); T2DM-*B.t*-EV (T2DM rats gavaged with *B.t*-EV); T2DM-P*B.t* (T2DM rats gavaged with P*B.t)*.

Histopathological examination revealed that the pancreatic islet structure and the histological features of islet cells were comparable in the NC-PBS, NC-P*B.t, *and NC-*B.t*-EVs rats. No specific changes were observed under the microscope (Figure 9a, b, c(Fig. 9)). In T2DM-PBS rats, there was an increase in the number of islets and changes in islet cells. These included atrophy, vocalization, and necrosis, as shown in Figure 9d(Fig. 9). In the T2DM-P*B.t *rats, islet atrophy and vacuolization were observed in the islet cells (Figure 9e(Fig. 9)). In the T2DM-*B.t*-EVs rats, the structure and organization of islets tended to be normal. The severity of the observed changes in islet cells was milder than in the other two groups (Figure 9f(Fig. 9)). 

More results are provided in the Supplementary Data. 

## Discussion

The current study was designed to assess the effects of *B.t *derivatives (P*B.t *and *B.t*-EVs) in an HFD/STZ-induced T2DM model in Wistar rats. We evaluated their effect on obesity indices, metabolic parameters, gene expression related to diabetes and inflammation, and gut microbiota composition. We found that intervention with P*B.t *and *B.t*-EVs significantly ameliorated metabolic dysregulation. This was achieved by reducing obesity indices, enhancing insulin sensitivity, improving glucose tolerance, and affecting lipid profile, including lowering TG and TC. Both treatments also regulated gene expression related to diabetes and inflammation. Additionally, we observed modulatory effects on gut microbiota targets.

According to new research, gut microbiota dysbiosis may play a crucial role in the onset and progression of T2DM. Consequently, modifying the gut microbiota through dietary changes, including postbiotic and paraprobiotic supplementation, is therefore proposed to be a novel and promising strategy for managing T2DM (Cabello-Olmo et al., 2021[6], Sionek and Gantner, 2025[41]). However, due to safety concerns associated with the administration of live probiotics, particularly the risks of infection and horizontal gene transfer, the use of inactivated cells or their components has been considered a safer and more practical alternative, especially for vulnerable populations such as immunocompromised individuals (Teame et al., 2020[49]). Within the gut microbiota, *Bacteroides* is sometimes the most abundant genus; however, it is also the most variable (Liu et al., 2023[28]). To address this variability and utilize the NGP's potential of *B.t*, we selected its derivatives, P*B.t *and *B.t*-EVs, which have shown beneficial effects in previous studies both in vitro and in vivo, including modulation of nutrient metabolism, immune homeostasis, and mucosal barrier function (Vaezijoze et al., 2025[51], Zheng et al., 2023[61]). Notably, *B.t* allocates approximately 20 % of its genome to polysaccharide utilization loci, enabling it to degrade a wide variety of dietary polysaccharides (Wong et al., 2024[53]). These characteristics may confer a selective advantage within the dynamic and competitive gut environment. As a result, certain derivatives of *B.t* are promising candidates for the targeted modulation of host-microbe interactions. Given the potential safety and stability benefits of non-viable microbial components over live bacteria, we focused on P*B.t *and *B.t*-EVs as functional candidates. To our knowledge, this is the first study to evaluate the potential effects of these microbial products in a T2DM rat model. We investigated their effect on glucose homeostasis, the expression of inflammation-related genes, and the modulation of host metabolic signaling pathways. These insights contribute to the evolving understanding of microbial-derived interventions for managing of metabolic disorders.

Numerous animal studies have demonstrated that T2DM increases obesity indices, while various probiotics have been shown to mitigate these effects (Bagheripour et al., 2024[4], Chen et al., 2018[7], Hasanian-Langroudi et al., 2024[19]). As expected, rats in the T2DM-PBS rats exhibited significant increases in BW, BMI, and Lee index compared to the NC-PBS rats, indicating the development of obesity-associated features in diabetic conditions. Treatment with *B.t*-EVs resulted in a significant reduction of these obesity indices in diabetic rats. Although treatment with P*B.t *also reduced obesity indices, this decrease did not reach statistical significance. Vaezijoze S et al. demonstrated that *B.t*-EVs enhance proprotein convertase subtilisin/kexin type 1(PCSK1) gene expression, while P*B.t* increases glucagon like-peptide 1 receptor (GLP-1) gene expression, both of which contribute to obesity management. They proposed these derivatives as postbiotics and paraprobiotics (Vaezijoze et al., 2025[51]). 

Our findings demonstrated that T2DM-PBS rats exhibited increased glycemic indices relative to NC-PBS rats, which is consistent with previous studies (Sah et al., 2016[38], Szkudelski, 2012[43]). Treatment with P*B.t *and *B.t*-EVs both reduced insulin levels, improved OGTT, and decreased HOMA-IR, indicating reversal of these alterations. Notably, only *B.t*-EVs treatment resulted in a statistically significant reduction in FBG. Although lowered FBG was also observed in the P*B.t-*treated rats, this reduction was not statistically significant. This superiority may be attributed to the nanoscale size of *B.t*-EVs, which enables them to cross the intestinal barrier, reach distant organs such as the liver, and directly interact with epithelial and immune cells, thereby exerting stronger systemic metabolic regulatory effects (Ashrafian et al., 2021[2], Jones et al., 2020[20]). Consistent with previous reports, our data suggest that dyslipidemia contributes to diabetes development (Kumari et al., 2023[22], Tangvarasittichai, 2015[47]). Both treatments also led to a significant decrease in TG and TC, along with a non-significant but evident decrease in LDL-C levels. In line with our own findings, supplementation with P*B.t *in HFD mice reduced glycemia, improved glucose tolerance, and lowered circulating lipopolysaccharide-binding protein (LBP) and IL-6. Together, these findings suggest that P*B.t *confers metabolic benefits by attenuating systemic inflammation and reinforcing the intestinal barrier. Importantly, our results also indicate that non-viable microbial preparations may serve as effective adjuncts for improving metabolic health (Lee et al., 2024[25]). In comparison, a significant increase in HDL-C was observed only in *B.t*-EVs-T2DM rats. Given the limited available data on *B.t*-EVs in this context, relevant insights were inferred from studies on *A. muciniphila*, a recognized NGP effective in T2DM, which shares a similar mucin-degrading mechanism with *B.t* (Glover et al., 2022[16], Lalowski and Zielińska, 2024[24]). Consistent with our findings, previous study have reported that *A. muciniphila,* along with its derivatives and pasteurized forms, can improve FBG, insulin, and lipid profile in T2DM (Deng et al., 2022[11]). Similarly, Ashrafian et al. in another study showed that pasteurized *A. muciniphila* and its EVs significantly reduce FBG and lipid profile in obese mice, further supporting their potential as effective paraprobiotic and postbiotic agents (Ashrafian et al., 2021[2]). 

Previous studies have shown that P*B.t* and *B.t*-EVs regulate the expression of several critical metabolic genes, indicating that even non-viable bacteria can influence metabolic pathways through structural elements and residual metabolites (Vaezijoze et al., 2025[51]). In this context, our results showed that treatment with P*B.t *and *B.t*-EVs downregulated gene expression of CB1, IL-1β, and IL-6, but upregulated CB2, PI3K, Akt, IL-4, and IL-10. These changes suggest enhanced insulin signaling and a shift toward an anti-inflammatory profile, which may underlie the observed improvements in metabolism. In line with our findings, a recent study in a dextran sulfate sodium (DSS)-induced colitis mouse model, *B.t*- EVs increased IL-10 and decreased IL-6 expression, highlighting their anti-inflammatory potential in vivo (Fonseca et al., 2022[12]). In the same disease model, Yinhe et al. study further supported our findings by showing that P*B.t *significantly downregulated colonic IL-6, highlighting that even non-viable bacteria can exert local anti-inflammatory effects through modulation of cytokine signaling (Yinhe et al., 2024[57]). In line with our results achieved through treatment with P*B.t* and *B.t*-EVs, Plovier et al. demonstrated that both pasteurized *A. muciniphila* and its EVs significantly downregulated CB1 expression and enhanced Akt activation in the liver of HFD-induced T2DM mice, supporting their role in modulating metabolic signaling pathways (Plovier et al., 2017[35]). In addition, recent evidence from a zebrafish model of T2DM demonstrated that pasteurized *A. muciniphila* modulated inflammation by downregulating IL-6 and upregulating IL-4 (Qu et al., 2025[36]). While our data demonstrated anti-inflammatory and metabolic benefits of P*B.t *and *B.t*-EVs, EVs derived from *Bacteroides uniformis* have been shown to induce M1 macrophage polarization and exacerbate gut inflammation in weaning mice (Tang et al., 2024[46]), thereby underscoring potential strain-specific differences within the *Bacteroides* genus.

Considering the role of PI3K/Akt in insulin signaling and glucose regulation, as well as the involvement of ECS alterations and cytokine networks in T2DM progression and inflammation, the upregulation of PI3K/Akt signaling and modulation of pro- and anti-inflammatory cytokine gene expression may contribute to metabolic improvements. The gut microbiota and ECS, through shared signaling pathways, play a role in modulating inflammation. Microbiota-derived compounds, particularly those from the *Bacteroidetes* phylum, can alter the levels of CB1, which is involved in regulating energy balance and inflammatory responses in peripheral tissues, and CB2, which is primarily linked to immune regulation. These receptors, along with the activity of enzymes involved in the production and degradation of regulatory lipids, are influenced by microbial factors. These effects are accompanied by the activation of the PI3K/Akt pathway in the liver, which is involved in regulating cellular responses to inflammation as well as in insulin signaling and maintaining cellular sensitivity to it. This pathway suppresses the translocation of nuclear factor kappa B (NF-κB) and prevents the activation of the nucleotide-binding domain and leucine-rich repeat protein 3 (NLRP3) inflammasome. The NLRP3 inflammasome is an intracellular sensor of damage-associated signals that initiates immune responses by producing inflammatory cytokines, thereby reducing the expression of IL-1β and IL-6. IL-1β is a major inducer of acute inflammation, while IL-6 contributes to the persistence of chronic inflammation and the development of IR. In contrast, the upregulation of IL-10 and IL-4 helps suppress inflammation and restore immune balance. This microbiota-endocannabinoid axis, through shared cellular signals, contributes to the control of inflammation and disorders resulting from IR. In this context, within the gut-liver axis, microbial components, particularly LPS, can cross the epithelial barrier and activate immune receptors, leading to inflammation and IR, a process interlinked with ECS, HFD, and altered microbiota composition (Pietro et al., 2013[34], Vasincu et al., 2023[52]).

Given the relationship between T2DM and gut microbiota, the current study assessed the influence of P*B.t *and *B.t*-EVs on microbial composition. The study targeted bacterial taxa selected for their relevance to T2DM and their potential interactions with *B.t*. Significant changes in the abundance of targeted bacteria were observed. Previous studies have reported that T2DM markedly alters gut microbiota composition. This is characterized by a significant increase in copy numbers of *Firmicutes, Proteobacteria*, and *Actinobacteria*, accompanied by a reduction in *Bacteroidetes* (Craciun et al., 2022[9], Upadhyaya and Banerjee, 2015[50]). We found that treatment with P*B.t *and *B.t*-EVs reversed these alterations. Both treatments notably reduced *Firmicutes* and *Actinobacteria*, while significantly restoring the abundance of *Bacteroidetes*. Moreover, the elevated *F/B* ratio in T2DM was significantly lowered by both interventions. At the genus level, P*B.t *and *B.t*-EVs treatment significantly increased beneficial bacteria, including *F. prausnitzii* and *B.t*. These bacteria were both diminished in T2DM-PBS. In line with our findings, P*B.t *in a DSS-induced colitis mouse model modulated gut microbiota by increasing the abundance of *Lactobacillus*, *Bacteroides*, and *F. prausnitzii*. This supports its potential role in restoring microbial balance (Yinhe et al., 2024[57]). Additionally, another study using a T2DM rat model demonstrated that both pasteurized *A. muciniphila *and its outer membrane protein modulated the gut microbiota. These interventions reduced the abundance of *Firmicutes* and increased the levels of *Bacteroidetes*. As a result, the *F/B* ratio decreased, indicating an improved microbial balance (Deng et al., 2022[11]). These findings further support the ability of non-viable microbial components to beneficially modulate gut microbiota composition. They also corroborate the metabolic benefits observed with P*B.t *and *B.t*-EVs in our study. In T2DM, gut dysbiosis is characterized by an increased abundance of *Proteobacteria* and *Actinobacteria*, both of which are enriched in LPS. Elevated LPS levels activate the toll-like receptor 4 (TLR4) and NF-κB signaling pathways. This increases the expression of IL-1β and IL-6, which promote systemic inflammation and IR (Wu et al., 2023[54]). Simultaneously, short-chain fatty acid (SCFA)-producing bacteria such as *F. prausnitzii*, *Clostridium cluster IV*, and *A. muciniphila *are significantly reduced in T2DM. This impairs butyrate-mediated activation of G protein-coupled receptor 109A (GPR109A) and free fatty acid receptor 2 (FFAR2). As a result, gut barrier integrity weakens, and the secretion of GLP-1 and peptide YY (PYY) decreases, exacerbating metabolic dysfunction (Gurung et al., 2020[18], Liu et al., 2023[29], Wu et al., 2023[54]). Furthermore, an elevated *F/B *ratio commonly observed in T2DM, is linked to increased energy harvest and chronic low-grade inflammation (Letchumanan et al., 2022[26]). It is also associated with decreased levels of propionate and acetate. These metabolites modulate hepatic gluconeogenesis and appetite, further linking dysbiosis to hyperglycemia and obesity (Magne et al., 2020[31]). *A. muciniphila* and *B.t* promote mucin degradation and maintain gut barrier function through enzymatic activity (Glover et al., 2022[16]). These microbiota alterations collectively exacerbate metabolic inflammation and impair glucose homeostasis in T2DM.

Collectively, in the current study, we demonstrated that interventions P*B.t *and *B.t*-EVs improved obesity indices, glycemic indices, and lipid profile. These interventions also enhanced the expression of genes associated with diabetes and inflammation. Additionally, they targeted gut bacteria composition. This provides a comprehensive evaluation of their effects on multiple aspects of T2DM, linking microbiota composition with host inflammatory status and metabolic signaling. The present work advances our understanding of nonviable microbial components as modulators of metabolic health. It lays a foundation for subsequent clinical evaluation and therapeutic development. The study's translational relevance is underscored by the use of nonviable modalities that offer favorable safety and stability profiles, supporting potential clinical applicability. One possible explanation for the beneficial effects of pasteurized forms and EVs may lie in the preservation of bioactivity in structural components, such as membrane proteins, which remain stable after inactivation and continue to engage with host signaling pathways. 

Despite encouraging results and their potential clinical relevance, this study has certain limitations. The primary limitation arises from inherent differences in microbiota composition and immune system function between rats and humans, which warrants caution when extrapolating the findings. Moreover, we did not analyze the contents of EVs derived from *B.t*, limiting our ability to fully elucidate the mechanisms through which these bacterial components may improve glucose metabolism and insulin sensitivity. Additional investigations are required to validate the beneficial effects of *B.t *derivatives in the management of T2DM in humans and to assess possible risks or adverse outcomes. Furthermore, studies using germ-free mice are recommended to confirm the observed effects.

## Conclusion

This study highlights the beneficial potential of P*B.t *and *B.t*-EVs in managing T2DM by improving metabolic parameters, modulating inflammatory responses, and restoring gut microbiota balance. These findings suggest that non-viable microbial components may serve as safer alternatives to live probiotics and could be effective candidates for managing metabolic disorders. Further investigations encompassing broader molecular pathways and clinical validations are warranted to fully realize the translational potential of these promising therapies.

## Notes

Seyed Davar Siadat and Maryam Tohidi (Prevention of Metabolic Disorders Research Center, Research Institute for Metabolic and Obesity Disorders, Research Institute for Endocrine Sciences, Shahid Beheshti University of Medical Sciences, Tehran, Iran; P.O. Box 19395-4763 Tehran, Islamic Republic of Iran, Phone: 98 21 22409301-5, Fax: 98 21 22402463, E-mail: tohidi@endocrine.ac.ir) contributed equally as corresponding author.

## Declaration

### Ethics approval statement

All experimental procedures conducted on rats received approval from the Ethics Committee of the Research Institute for Endocrine Sciences, Shahid Beheshti University of Medical Sciences (Ethic Code: IR.SBMU.ENDOCRINE.REC.1402.043).

### Funding

This study was not supported by any specific grant from funding bodies in the public, commercial, or non-profit sectors.

### Conflict of interest

All authors certify that they have no affiliations with or involvement in any organization or entity with any financial interest or non-financial interest in the subject matter or materials discussed in this manuscript.

### Data statement

All the data that support the findings of this study are available from the corresponding author, upon reasonable request.

### Using Artificial Intelligence (AI)

Authors used AI-assisted technologies to check for grammatical edits.

### CRediT authorship contribution statement

**Conceptualization: **FHL, SDS, AG, MT, **Methodology: **FHL, SDS, MH, AG, MT, **Validation: **SDS, MH, AG, MT,** Formal analysis: **FHL, AG, MH,** Investigation: **FHL,** Data Curation: **FHL, MT, **Writing-Original Draft: **FHL, **Writing-Review & Editing: **FHL, SDS, MH, AG, MT, **Visualization: **FHL, **Supervision: **SDS, MH, AG, MT, **Project administration**: SDS, MT.

### Acknowledgments

This work is based on the doctoral dissertation of Farzaneh Hasanian-Langroudi conducted at the Prevention of Metabolic Disorders Research Center, Research Institute for Metabolic and Obesity Disorders, affiliated with the Research Institute for Endocrine Sciences, Shahid Beheshti University of Medical Sciences, Tehran, Iran. The authors gratefully acknowledge the assistance of the laboratory personnel of the Research Institute for Endocrine Sciences, Shahid Beheshti University of Medical Sciences, Tehran, Iran, as well as the support provided by the Mycobacteriology and Pulmonary Research Department and the Microbiology Research Center at the Pasteur Institute of Iran, Tehran, Iran. 

## Supplementary Material

Supplementary information

Supplementary data

## Figures and Tables

**Table 1:**
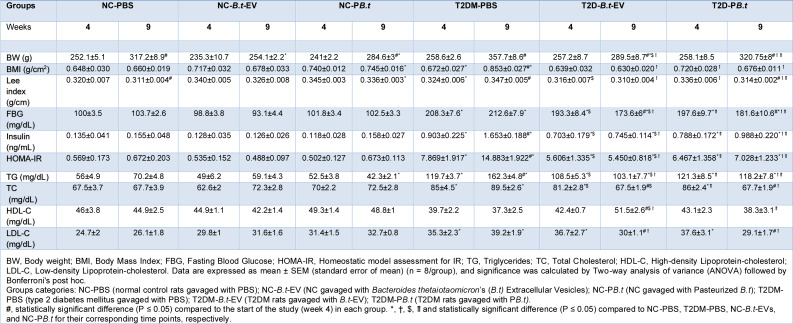
The effect of P*B.t *and *B.t*-EVs on obesity indices and biochemical parameters

**Figure 1:**
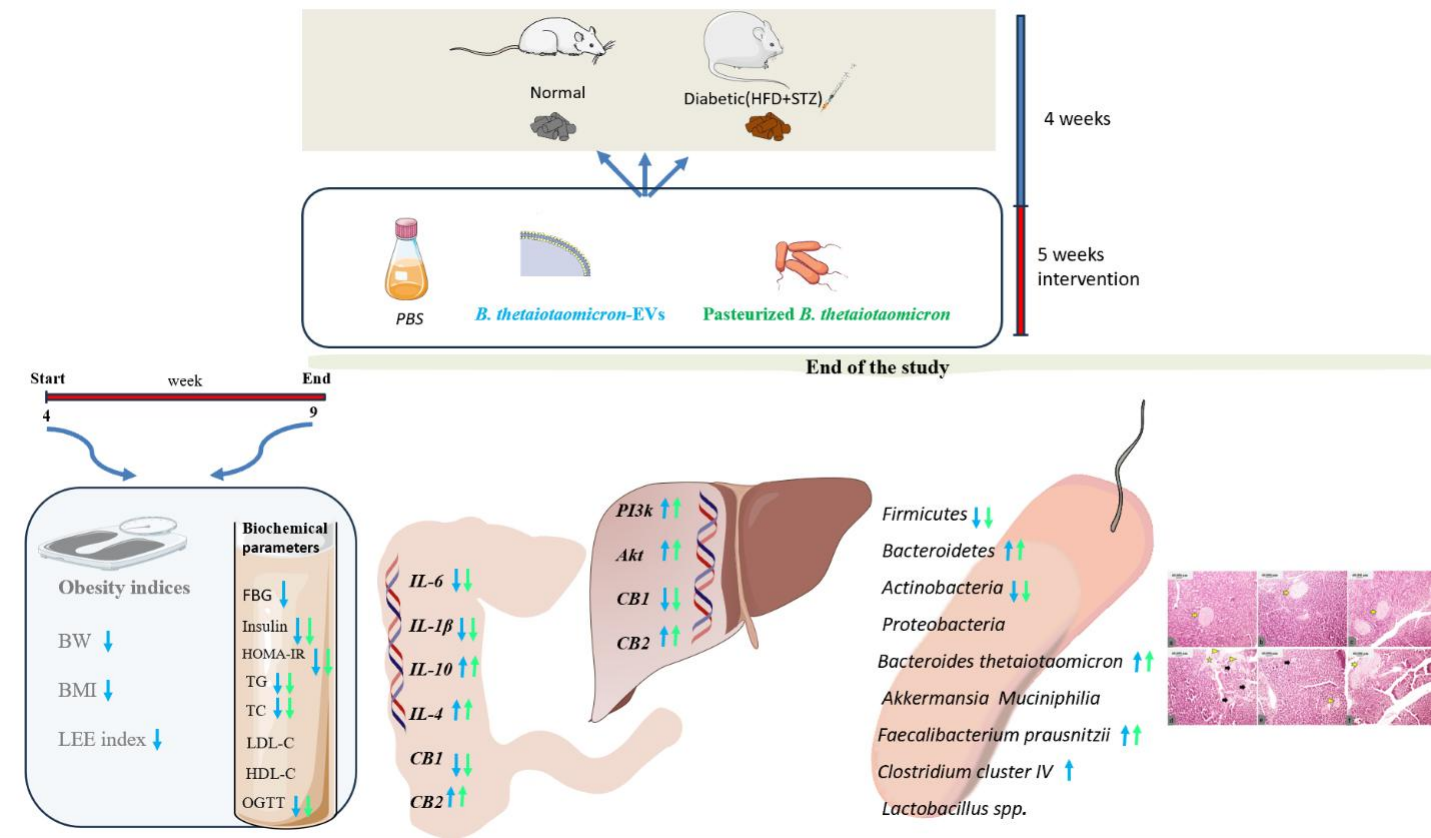
Graphical abstract
