# Supplementary material for: Pasteurized Bacteroides thetaiotaomicron and its extracellular vesicles improve metabolic profiles, expression of genes associated with diabetes and inflammation, and gut microbiota in type 2 diabetic rats
Source: EXCLI J. 2025 Dec 4;24:1743–66. doi: 10.17179/excli2025-8860 (PMC12853027; doi:10.17179/excli2025-8860)
Supplement: Supplementary information [file EXCLI-24-1743-s-001.pdf]

EXCLI Journal 2025;24:1743-1766 – ISSN 1611-2156
Received: August 23, 2025, accepted: November 14, 2025, published: December 04, 2025

Supplementary information to:

Original article:

PASTEURIZED *BACTEROIDES THETAIOTAOMICRON* AND ITS EXTRACELLULAR VESICLES IMPROVE METABOLIC PROFILES, EXPRESSION OF GENES ASSOCIATED WITH DIABETES AND INFLAMMATION, AND GUT MICROBIOTA IN TYPE 2 DIABETIC RATS

Farzaneh Hasanian-Langroudi[1], Mehdi Hedayati[2], Asghar Ghasemi[3], Seyed Davar Siadat[4,5*], Maryam Tohidi[1*]

[1] Prevention of Metabolic Disorders Research Center, Research Institute for Metabolic and Obesity Disorders, Research Institute for Endocrine Sciences, Shahid Beheshti University of Medical Sciences, Tehran, Iran
[2] Cellular and Molecular Endocrine Research Center, Research Institute for Endocrine Molecular Biology, Research Institute for Endocrine Sciences, Shahid Beheshti University of Medical Sciences, Tehran, Iran
[3] Endocrine Physiology Research Center, Research Institute for Endocrine Molecular Biology, Research Institute for Endocrine Sciences, Shahid Beheshti University of Medical Sciences, Tehran, Iran
[4] Department of Mycobacteriology and Pulmonary Research, Pasteur Institute of Iran, Tehran, Iran
[5] Microbiology Research Center, Pasteur Institute of Iran, Tehran, Iran

* **Corresponding authors:** Maryam Tohidi, Prevention of Metabolic Disorders Research Center, Research Institute for Metabolic and Obesity Disorders, Research Institute for Endocrine Sciences, Shahid Beheshti University of Medical Sciences, Tehran, Iran. P.O. Box 19395-4763Tehran, Islamic Republic of Iran, Phone: 98 21 22409301-5, Fax: 98 21 22402463, E-mail: tohidi@endocrine.ac.ir
Seyed Davar Siadat, Department of Mycobacteriology and Pulmonary Research, Pasteur Institute of Iran, Tehran, Iran; Microbiology Research Center, Pasteur Institute of Iran, Tehran, Iran. P.O. Box 13169-4355Tehran, Islamic Republic of Iran, Phone: 98 21 64112823, E-mail: d.siadat@gmail.com

https://dx.doi.org/10.17179/excli2025-8860

This is an Open Access article distributed under the terms of the Creative Commons Attribution License (https://creativecommons.org/licenses/by/4.0/).

EXCLI Journal 2025;24:1743-1766 – ISSN 1611-2156
Received: August 23, 2025, accepted: November 14, 2025, published: December 04, 2025

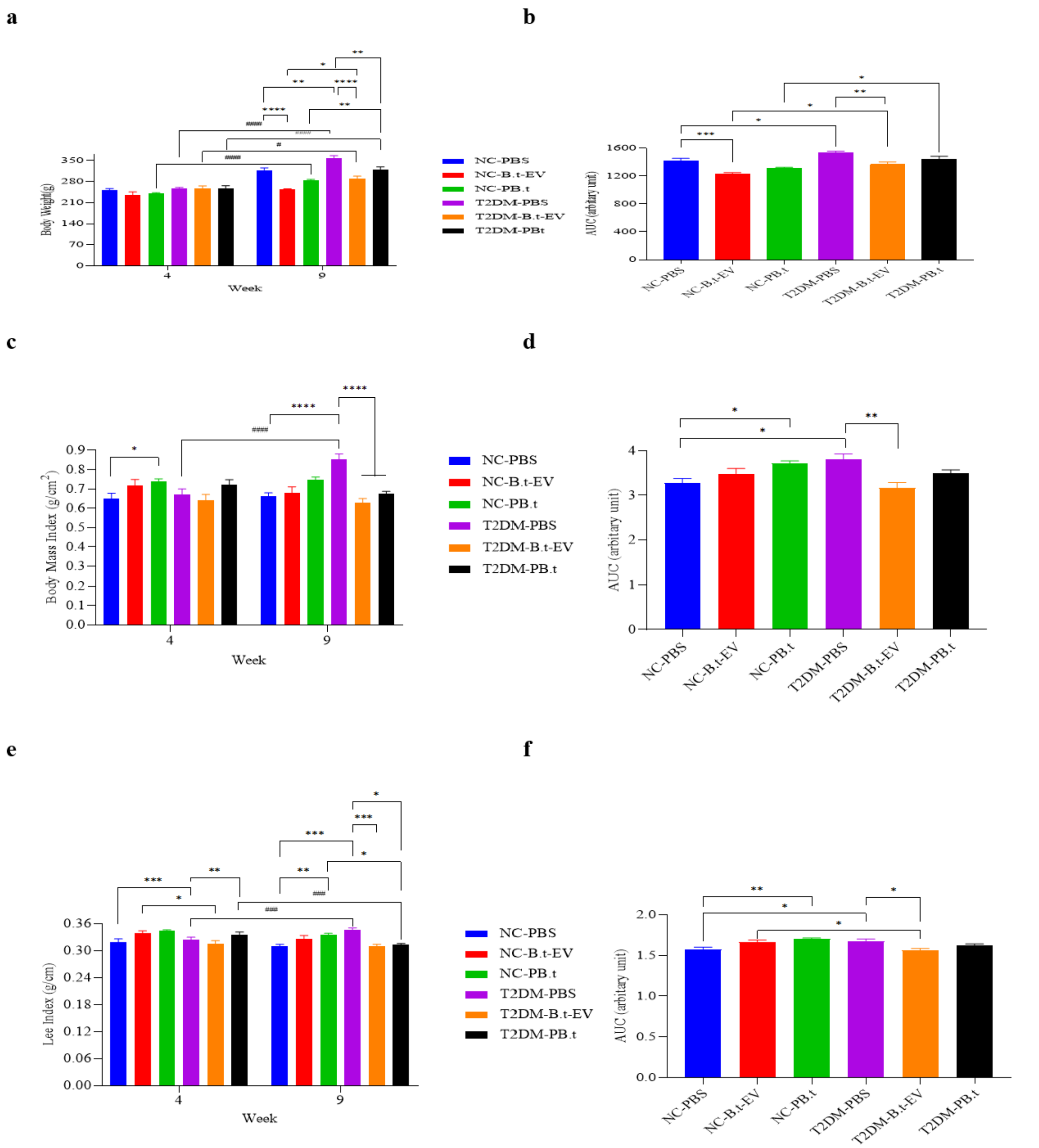

Supplementary Figure 1: The effect of P*B.t* and *B.t*-EVs on obesity indices. (a) BW (Body Weight), and (b) its AUC (the area under the curves), (c) BMI (body mass index), and (d) its AUC, (e) Lee index, and (f) its AUC. Data are expressed as mean ± SEM (standard error of mean) (n = 8/group); *, $P < 0.05$; **, $P < 0.01$; ***, $P < 0.001$; ****, $P < 0.0001$ by post hoc Bonferroni's Two-way analysis of variance (ANOVA). #, statistically significant difference compared to the start of the study (week 4) in each group. *, statistically significant differences between groups in weeks 4 and 9.

Groups categories: NC-PBS (normal control rats gavaged with PBS); NC-*B.t*-EV (NC gavaged with *Bacteroides thetaiotaomicron's* Extracellular Vesicles); NC-P*B.t* (NC gavaged with Pasteurized *B.t*); T2DM-PBS (type 2 diabetes mellitus gavaged with PBS); T2DM-*B.t*-EV (T2DM rats gavaged with *B.t*-EV); T2DM-P*B.t* (T2DM rats gavaged with P*B.t).*

EXCLI Journal 2025;24:1743-1766 – ISSN 1611-2156
Received: August 23, 2025, accepted: November 14, 2025, published: December 04, 2025

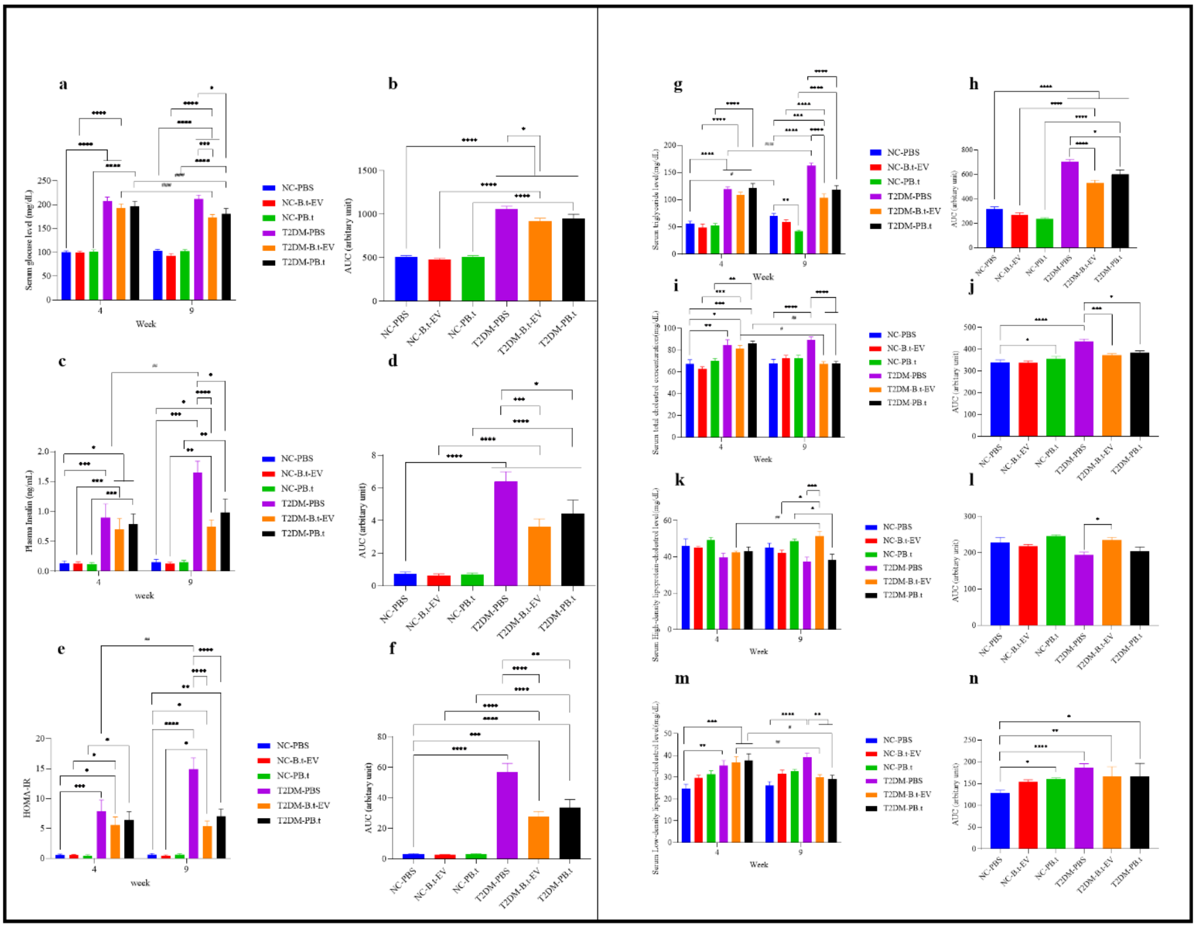

Supplementary Figure 2: The effect of P*B.t* and *B.t*-EVs on glycemic indices and lipid profile. Serum level of (a) FBG (Fast blood Glucose), and (b) its AUC (the area under the curve), (c) Insulin and (d) its AUC, (e) HOMA-IR and (f) its AUC, (g) TG (Triglyceride) and (h) its AUC, (i) TC (total cholesterol level and (J) its AUC, (k) HDL (high-density lipoprotein-cholesterol) and (l) its AUC, (m) LDL-C (low-density lipoprotein- cholesterol) and (n) its AUC.

Data are expressed as mean ± SEM (standard error of mean) (n = 8/group), *, $P < 0.05$; **, $P < 0.01$; ***, $P < 0.001$; ****, $P < 0.0001$ by post hoc Bonferroni's Two-way analysis of variance (ANOVA). #, statistically significant difference compared to the start of the study (week 4) in each group. *, statistically significant differences between groups in weeks 4 and 9.

Groups categories: NC-PBS (normal control rats gavaged with PBS); NC-*B.t*-EV (NC gavaged with *Bacteroides thetaiotaomicron*'s Extracellular Vesicles); NC-P*B.t* (NC gavaged with Pasteurized *B.t*); T2DM-PBS (type 2 diabetes mellitus gavaged with PBS); T2DM-*B.t*-EV (T2DM rats gavaged with *B.t*-EV); T2DM-P*B.t* (T2DM rats gavaged with P*B.t)*.

EXCLI Journal 2025;24:1743-1766 – ISSN 1611-2156
Received: August 23, 2025, accepted: November 14, 2025, published: December 04, 2025

Supplementary Table 1: Composition of the High-Fat Diet used in the study

materials	amount
powdered normal-pellet diet	1000 g
butter	531 g
casein	125 g
DL-methionine	3 g
vitamin mix	7 g
mineral mix	42 g

Supplementary Table 2: Sequences of Primers Used in the Reaction of qPCR

Name		Seq 5'……. 3'	Primer length (bp)	Product length (bp)	Accession number
Pik3cg	F	TGACAGGCACAACGACAACA	20	165	XM_006240004.5
	R	TTGCCCGAAGTTCCCATCACA	21		
Akt1	F	CTCCTCAAGAATGATGGCACCT	22	217	XM_039111773.2
	R	TCCACTCTTCCCGCTCCT	18		
Cnr1	F	GTTGACTTCCATGTATTCCAC-CGTA	25	125	XM_063287167.1
	R	ATGTACCTGTCGATGGCTGTGA	22		
Cnr2	F	CAGCGTGACCATGACCTTC	19	185	XM_063288330.1
	R	TCCACCCCATGAGCGGTAG	19		
IL6	F	CTTCCTACCCCAACTTCCAATG	22	135	NM_012589.2
	R	CCGAGTAGACCTCATAGTGACC	22		
IL10	F	CCAGTTCTTTCCCCTGTAGCCA	22	152	XM_008769426.4
	R	TCGCCCAGAGACAGACAAGCAA	22		
IL1b	F	TACCTATGTCTTGCCCGTGGA	21	122	NM_031512.2
	R	ATCACACACTAGCAGGTCGTC	21		
IL4	F	ATGCACCGAGATGTTTGTAC-CAGA	24	169	NM_201270.1
	R	ACAGAGTTTCCTCAGTTCACCG	22		
Eef2	F	GGTCCCAACATTCTCACCGACA	22	140	NM_017245.2
	R	ACATCAAATCGCACACCACGCA	22		

EXCLI Journal 2025;24:1743-1766 – ISSN 1611-2156
Received: August 23, 2025, accepted: November 14, 2025, published: December 04, 2025

Supplementary Table 3: Sequences of Primers Used in the Reaction of qPCR

Micro-organism		Primer Sequence (5'-3')	Primer length (bp)	Product size (bp)	References
Part A: Phylum					
Actino-bacteria	F	CGCGGCCTATCAGCTTGTTG	20	643	Bredholt et al., 2008
	R	CCGTACTCCCCAGGCGGGG	20		
Proteo-bacteria	F	CATGACGTTACCCGCAGAAGAAG	23	195	Murri et al., 2013
	R	CTCTACGAGACTCAAGCTTGC	21		
Bactero-idetes	F	GTTTAATTCGATGATACGCGAG	22	211	Matsuki et al., 2004
	R	TTAASCCGACACCTCACGG	19		
Firmicutes	F	GCAGTAGGGAATCTTCCG	18	181	Senkovs et al., 2021
	R	ATTACCGCGGCTGCTGG	17		
Part B: Genus, Species					
Akkermansia Muciniphila	F	CAGCACGTGAAGGTGGGGAC	20	329	Schnee-berger et al., 2015
	R	CCTTGCGGTTGGCTTCAGAT	20		
Bacteroides thetaiotaomi-cron	F	GAGGAAGGTCCCCCACATTG	20	298	In this study
	R	ACCCATAGGGCAGTCATCCT	20		
Lacto-bacillus spp.	F	AGCAGTAGGGAATCTTCCA	19	341	Alioua et al., 2016
	R	ATTYCACCGCTACACATG	18		
Clostridium Cluster IV	F	ACAATAAGTAATCCACCTGG	20	298	Hermann-Bank et al., 2013
	R	CTTCCTCCGTTTTGTCAA	18		
Faecali-bacterium Prausnitzii	F	GGAGGAAGAAGGTCTTCGG	19	248	Fitzgerald et al., 2018
	R	AATTCCGCCTACCTCTGCACT	21		
16s Universal	F	TCCTACGGGAGGCAGCAGT	19	348	Qian et al., 2018
	R	GGACTACCAGGG-TATCTAATCCTGTT	26		
E. coli	F	CATTGACGTTACCCGCAGAA-GAAGC	25	195	Bartosch et al., 2004
	R	CTCTACGAGACTCAAGCTTGC	21		

Alioua S, Abdi A, Fhoula I, Bringel F, Boudabous A, Ouzari IH. Diversity of vaginal lactic acid bacterial microbiota in 15 Algerian pregnant women with and without bacterial vaginosis by using culture independent method. Journal of Clinical and Diagnostic Research: JCDR. 2016;10(9):DC23.

Bartosch S, Fite A, Macfarlane GT, McMurdo ME. Characterization of bacterial communities in feces from healthy elderly volunteers and hospitalized elderly patients by using real-time PCR and effects of antibiotic treatment on the fecal microbiota. Applied and environmental microbiology. 2004;70(6):3575-81.

Bredholt H, Fjærvik E, Johnsen G, Zotchev SB. Actinomycetes from sediments in the Trondheim fjord, Norway: diversity and biological activity. Marine drugs. 2008;6(1):12-24.

Fitzgerald CB, Shkoporov AN, Sutton TD, Chaplin AV, Velayudhan V, Ross RP, et al. Comparative analysis of Faecalibacterium prausnitzii genomes shows a high level of genome plasticity and warrants separation into new species-level taxa. BMC genomics. 2018;19:1-20.

Hermann-Bank ML, Skovgaard K, Stockmarr A, Larsen N, Mølbak L. The Gut Microbiotassay: a high-throughput qPCR approach combinable with next generation sequencing to study gut microbial diversity. BMC genomics. 2013;14:1-14.

Matsuki T, Watanabe K, Fujimoto J, Takada T, Tanaka R. Use of 16S rRNA gene-targeted group-specific primers for real-time PCR analysis of predominant bacteria in human feces. Applied and environmental microbiology. 2004;70(12):7220-8.

Murri M, Leiva I, Gomez-Zumaquero JM, Tinahones FJ, Cardona F, Soriguer F, et al. Gut microbiota in children with type 1 diabetes differs from that in healthy children: a case-control study. BMC medicine. 2013;11:1-12.

Qian Y, Yang X, Xu S, Wu C, Qin N, Chen S-D, et al. Detection of microbial 16S rRNA gene in the blood of patients with Parkinson's disease. Frontiers in aging neuroscience. 2018;10:156.

Schneeberger M, Everard A, Gómez-Valadés AG, Matamoros S, Ramírez S, Delzenne NM, et al. Akkermansia muciniphila inversely correlates with the onset of inflammation, altered adipose tissue metabolism and metabolic disorders during obesity in mice. Scientific reports. 2015;5(1):16643.

Senkovs M, Nikolajeva V, Makarenkova G, Petrina Z. Influence of Trichoderma asperellum and Bacillus subtilis as biocontrol and plant growth promoting agents on soil microbiota. Annals of Microbiology. 2021;71:1-10.
