# Supplementary material for: Pasteurized Bacteroides thetaiotaomicron and its extracellular vesicles improve metabolic profiles, expression of genes associated with diabetes and inflammation, and gut microbiota in type 2 diabetic rats
Source: EXCLI J. 2025 Dec 4;24:1743–66. doi: 10.17179/excli2025-8860 (PMC12853027; doi:10.17179/excli2025-8860)
Supplement: Supplementary data [file EXCLI-24-1743-s-002.pdf]

EXCLI Journal 2025;24:1743-1766 – ISSN 1611-2156
Received: August 23, 2025, accepted: November 14, 2025, published: December 04, 2025

Supplementary data to:

Original article:

PASTEURIZED *BACTEROIDES THETAIOTAOMICRON* AND ITS EXTRACELLULAR VESICLES IMPROVE METABOLIC PROFILES, EXPRESSION OF GENES ASSOCIATED WITH DIABETES AND INFLAMMATION, AND GUT MICROBIOTA IN TYPE 2 DIABETIC RATS

Farzaneh Hasanian-Langroudi[1], Mehdi Hedayati[2], Asghar Ghasemi[3], Seyed Davar Siadat[4,5*], Maryam Tohidi[1*]

[1] Prevention of Metabolic Disorders Research Center, Research Institute for Metabolic and Obesity Disorders, Research Institute for Endocrine Sciences, Shahid Beheshti University of Medical Sciences, Tehran, Iran
[2] Cellular and Molecular Endocrine Research Center, Research Institute for Endocrine Molecular Biology, Research Institute for Endocrine Sciences, Shahid Beheshti University of Medical Sciences, Tehran, Iran
[3] Endocrine Physiology Research Center, Research Institute for Endocrine Molecular Biology, Research Institute for Endocrine Sciences, Shahid Beheshti University of Medical Sciences, Tehran, Iran
[4] Department of Mycobacteriology and Pulmonary Research, Pasteur Institute of Iran, Tehran, Iran
[5] Microbiology Research Center, Pasteur Institute of Iran, Tehran, Iran

* **Corresponding authors:** Maryam Tohidi, Prevention of Metabolic Disorders Research Center, Research Institute for Metabolic and Obesity Disorders, Research Institute for Endocrine Sciences, Shahid Beheshti University of Medical Sciences, Tehran, Iran. P.O. Box 19395-4763Tehran, Islamic Republic of Iran, Phone: 98 21 22409301-5, Fax: 98 21 22402463, E-mail: tohidi@endocrine.ac.ir
Seyed Davar Siadat, Department of Mycobacteriology and Pulmonary Research, Pasteur Institute of Iran, Tehran, Iran; Microbiology Research Center, Pasteur Institute of Iran, Tehran, Iran. P.O. Box 13169-4355Tehran, Islamic Republic of Iran, Phone: 98 21 64112823, E-mail: d.siadat@gmail.com

https://dx.doi.org/10.17179/excli2025-8860

This is an Open Access article distributed under the terms of the Creative Commons Attribution License (https://creativecommons.org/licenses/by/4.0/).

EXCLI Journal 2025;24:1743-1766 – ISSN 1611-2156
Received: August 23, 2025, accepted: November 14, 2025, published: December 04, 2025

Supplementary Table 4: Raw data of anthropometric measures in each group before and after intervention. Raw data is related to Table 1 and Supplementary Figure 1(a, b, c, d, e, and f)

Group	rat ID	BW (g)			BMI (g/cm2)			Lee Index (g/cm)		
		week 4 (before intervention)	week 9 (after intervention)	AUC (arbitrary unit)	week 4 (before intervention)	week 9 (after intervention)	AUC (arbitrary unit)	week 4 (before intervention)	week 9 (after intervention)	AUC (arbitrary unit)
NC-PBS	1S	230	306	1340	0.605	0.662	3.168	0.314	0.313	1.568
	2S	263	300	1408	0.651	0.68	3.328	0.319	0.319	1.595
	3S	237	298	1338	0.657	0.676	3.333	0.326	0.318	1.61
	4S	272	353	1563	0.753	0.697	3.625	0.341	0.314	1.638
	5S	262	361	1558	0.655	0.746	3.503	0.32	0.324	1.61
	7S	251	317	1420	0.67	0.62	3.225	0.328	0.304	1.58
	8S	260	303	1408	0.474	0.574	2.62	0.274	0.29	1.41
	9S	242	300	1355	0.72	0.626	3.365	0.336	0.305	1.603
	Mean	252.1	317.2	1424	0.648	0.66	3.271	0.319	0.31	1.577
	SEM	5.1	8.9	31.91	0.029	0.018	0.106	0.007	0.003	0.024
	SD	14.6	25.3	90.25	0.083	0.052	0.3	0.02	0.01	0.07
	N	8	8	8	8	8	8	8	8	8
NC-*B.t*-EV	1A	225	254	1198	0.687	0.704	3.478	0.336	0.333	1.673
	2A	224	260	1210	0.654	0.72	3.435	0.328	0.336	1.66
	3A	212	263	1188	0.619	0.626	3.113	0.322	0.313	1.588
	4A	206	245	1128	0.636	0.716	3.38	0.328	0.338	1.665
	5A	216	255	1178	0.705	0.527	3.08	0.343	0.288	1.578
	6A	282	251	1333	0.842	0.569	3.528	0.358	0.3	1.645
	7A	283	247	1325	0.864	0.762	4.065	0.363	0.349	1.78
	9A	235	258	1233	0.725	0.796	3.803	0.343	0.354	1.743
	Mean	235.3	254.1	1224	0.716	0.677	3.485	0.34	0.326	1.667
	SEM	10.7	2.2	25.24	0.032	0.033	0.116	0.005	0.008	0.024
	SD	30.3	6.2	71.38	0.091	0.094	0.328	0.014	0.023	0.068
	N	8	8	8	8	8	8	8	8	8
NC-P*B.t*	1P	240	272	1280	0.672	0.68	3.38	0.329	0.324	1.633
	2P	238	280	1295	0.743	0.7	3.608	0.346	0.327	1.683
	3P	244	295	1348	0.762	0.817	3.948	0.349	0.35	1.748
	4P	242	288	1325	0.747	0.72	3.668	0.346	0.33	1.69
	6P	254	298	1380	0.784	0.745	3.823	0.352	0.334	1.715
	7P	235	280	1288	0.725	0.776	3.753	0.343	0.344	1.718
	8P	242	285	1318	0.764	0.75	3.785	0.35	0.337	1.718

	**9P**	233	279	1280	0.719	0.773	3.73	0.342	0.344	1.715
	**Mean**	241	284.6	1314	0.739	0.745	3.712	0.344	0.336	1.703
	**SEM**	2.2	3	12.68	0.012	0.015	0.059	0.002	0.003	0.012
	**SD**	6.4	8.7	35.85	0.034	0.044	0.168	0.007	0.009	0.034
	**N**	8	8	8	8	8	8	8	8	8
**T2DM-PBS**	**1S**	248	391	1598	0.652	0.978	4.075	0.322	0.366	1.72
	**2S**	256	320	1440	0.64	0.761	3.503	0.317	0.334	1.628
	**3S**	270	343	1533	0.612	0.778	3.475	0.308	0.333	1.603
	**4S**	259	353	1530	0.616	0.8	3.54	0.311	0.337	1.62
	**5S**	265	339	1510	0.774	0.939	4.283	0.347	0.367	1.785
	**6S**	250	389	1598	0.657	0.882	3.848	0.323	0.348	1.678
	**8S**	258	366	1560	0.614	0.83	3.61	0.311	0.341	1.63
	**9S**	263	361	1560	0.812	0.859	4.178	0.356	0.347	1.758
	**Mean**	258.6	357.7	1541	0.672	0.853	3.814	0.324	0.346	1.678
	**SEM**	2.6	8.6	18.21	0.027	0.027	0.116	0.006	0.004	0.024
	**SD**	7.4	24.4	51.5	0.077	0.076	0.327	0.017	0.013	0.069
	**N**	8	8	8	8	8	8	8	8	8
**T2DM-*B.t*-EV**	**1A**	284	268	1380	0.603	0.638	3.103	0.303	0.315	1.545
	**2A**	250	268	1295	0.693	0.608	3.253	0.332	0.307	1.598
	**3A**	235	282	1293	0.533	0.583	2.79	0.294	0.298	1.48
	**5A**	289	313	1505	0.723	0.592	3.288	0.331	0.309	1.565
	**6A**	275	338	1533	0.731	0.639	3.425	0.335	0.303	1.595
	**7A**	234	289	1308	0.557	0.625	2.955	0.301	0.308	1.523
	**9A**	225	269	1235	0.535	0.593	2.82	0.297	0.303	1.5
	**10A**	266	289	1388	0.737	0.76	3.743	0.338	0.339	1.693
	**Mean**	257.2	289.5	1367	0.639	0.629	3.172	0.316	0.31	1.562
	**SEM**	8.7	8.7	37.51	0.032	0.02	0.114	0.006	0.004	0.023
	**SD**	24.6	24.8	106.1	0.091	0.056	0.323	0.019	0.012	0.067
	**N**	8	8	8	8	8	8	8	8	8
**T2DM-P*B.t***	**1P**	280	317	1493	0.7	0.699	3.498	0.327	0.32	1.618
	**2P**	230	310	1350	0.751	0.64	3.478	0.35	0.308	1.645
	**4P**	250	310	1400	0.772	0.671	3.608	0.35	0.315	1.663
	**5P**	250	296	1365	0.772	0.704	3.69	0.35	0.325	1.688
	**6P**	267	337	1510	0.74	0.666	3.515	0.339	0.309	1.62
	**7P**	296	350	1615	0.74	0.723	3.658	0.333	0.32	1.633
	**9P**	267	352	1548	0.747	0.665	3.53	0.341	0.307	1.62
	**10P**	225	294	1298	0.535	0.636	2.928	0.297	0.309	1.515
	**Mean**	258.1	320.7	1447	0.719	0.675	3.488	0.335	0.314	1.625
	**SEM**	8.5	8	38.97	0.02	0.01	0.084	0.006	0.002	0.017
	**SD**	24.1	22.8	110.2	0.077	0.03	0.239	0.017	0.006	0.05

EXCLI Journal 2025;24:1743-1766 – ISSN 1611-2156
Received: August 23, 2025, accepted: November 14, 2025, published: December 04, 2025

	**N**	8	8	8	8	8	8	8	8	8

BW, Body Weight; BMI, Body Mass Index; AUC, Area Under the Curve; NC-PBS, normal control rats gavaged with PBS; NC-*B.t*-EV, normal control rats gavaged with *Bacteroides thetaiotaomicron*'s (*B.t*) Extracellular Vesicles; NC-P*B.t*, normal control rats gavages with Pasteurized *B.t*; T2DM-PBS, type 2 diabetes mellitus gavaged with PBS; T2DM-*B.t*-EV, type 2 Diabetes mellitus gavaged with *B.t* EV; T2DM-P*B.t*, type 2 diabetes mellitus gavages with P*B.t*; SEM, standard error of mean; SD, standard deviation; N, number of rats.

EXCLI Journal 2025;24:1743-1766 – ISSN 1611-2156
Received: August 23, 2025, accepted: November 14, 2025, published: December 04, 2025

Supplementary Table 5: Raw data of FBG, Insulin and HOMA-IR in each group before and after intervention. Raw data is related to Table 1 and Supplementary Figure 2(a, b, c, d, e, and f)

Group	rat ID	FBG (mg/dL)			Insulin (ng/mL)			HOMA-IR		
		week 4 (before intervention)	week 9 (after intervention)	AUC (arbitrary unit)	week 4 (before intervention)	week 9 (after intervention)	AUC (arbitrary unit)	week 4 (before intervention)	week 9 (after intervention)	AUC (arbitrary unit)
NC-PBS	1S	105	104	522.5	0.12	0.06	0.45	0.518	0.281	1.996
	2S	113	114	567.5	0.06	0.09	0.375	0.279	0.431	1.775
	3S	85	92	442.5	0.04	0.23	0.675	0.138	0.885	2.559
	4S	92	100	480	0.05	0.37	1.05	0.180	1.582	4.405
	5S	112	108	550	0.18	0.33	1.275	0.829	1.483	5.781
	7S	103	113	540	0.25	0.04	0.725	1.061	0.200	3.151
	8S	94	98	480	0.03	0.07	0.25	0.118	0.282	1
	9S	96	101	492.5	0.35	0.05	1	1.431	0.230	4.152
	Mean	100	103.8	509.4	0.135	0.155	0.725	0.569	0.672	3.102
	SEM	3.505	2.678	15.01	0.041	0.048	0.128	0.173	0.203	0.562
	SD	9.914	7.573	42.46	0.116	0.135	0.361	0.49	0.575	1.589
	N	8	8	8	8	8	8	8	8	8
NC-*B.t*-EV	1A	83	102	462.5	0.04	0.14	0.45	0.139	0.613	1.879
	2A	100	115	537.5	0.29	0.16	1.125	1.226	0.755	4.952
	3A	84	105	472.5	0.11	0.06	0.425	0.394	0.259	1.632
	4A	106	91	492.5	0.16	0.07	0.575	0.695	0.254	2.374
	5A	103	85	470	0.25	0.08	0.825	1.092	0.294	3.465
	6A	97	80	442.5	0.11	0.17	0.7	0.431	0.562	2.481
	7A	102	84	465	0.03	0.27	0.75	0.142	0.953	2.739
	9A	116	83	497.5	0.03	0.06	0.225	0.162	0.211	0.933
	Mean	98.88	93.13	480	0.128	0.126	0.634	0.535	0.488	2.557
	SEM	3.893	4.486	10.23	0.035	0.026	0.099	0.152	0.097	0.435
	SD	11.01	12.69	28.94	0.1	0.074	0.279	0.43	0.275	1.23
	N	8	8	8	8	8	8	8	8	8
NC-P*B.t*	1P	105	100	512.5	0.04	0.04	0.2	0.155	0.191	0.865
	2P	85	98	457.5	0.19	0.18	0.925	0.676	0.736	3.529
	3P	114	111	562.5	0.19	0.26	1.125	0.915	1.205	5.298
	4P	111	108	547.5	0.20	0.08	0.7	0.921	0.375	3.24
	6P	102	119	552.5	0.19	0.14	0.825	0.805	0.684	3.722
	7P	91	89	450	0.04	0.25	0.725	0.157	0.922	2.696
	8P	101	96	492.5	0.04	0.12	0.4	0.179	0.490	1.672

EXCLI Journal 2025;24:1743-1766 – ISSN 1611-2156
Received: August 23, 2025, accepted: November 14, 2025, published: December 04, 2025

	**9P**	106	99	512.5	0.05	0.19	0.6	0.209	0.786	2.487
	**Mean**	101.9	102.5	510.9	0.118	0.158	0.688	0.502	0.673	2.939
	**SEM**	3.435	3.375	15.02	0.028	0.027	0.103	0.127	0.113	0.478
	**SD**	9.717	9.547	42.49	0.08	0.078	0.292	0.358	0.32	1.351
	**N**	8	8	8	8	8	8	8	8	8
**T2DM-PBS**	**1S**	201	200	890	0.40	2.18	6.45	3.421	18.303	54.31
	**2S**	176	180	1003	0.40	0.90	3.25	2.909	6.749	24.14
	**3S**	227	235	1155	1.05	1.11	5.4	9.967	10.898	52.16
	**4S**	191	200	977.5	0.48	1.99	6.175	3.863	16.675	51.34
	**5S**	204	205	1023	1.88	1.68	8.9	16.102	14.419	76.3
	**6S**	198	202	1000	1.81	1.13	7.35	14.994	9.567	61.4
	**8S**	238	242	1200	0.32	2.03	5.875	3.170	20.594	59.41
	**9S**	232	237	1173	0.88	2.20	7.7	8.527	21.864	75.98
	**Mean**	208.4	212.6	1053	0.903	1.653	6.388	7.869	14.88	56.88
	**SEM**	7.693	7.919	38.93	0.225	0.188	0.599	1.917	1.922	5.825
	**SD**	21.76	22.4	110.1	0.635	0.53	1.694	5.423	5.435	16.48
	**N**	8	8	8	8	8	8	8	8	8
**T2DM-*B.t*-EV**	**1A**	220	180	1000	0.46	0.61	2.675	4.233	4.573	22.02
	**2A**	203	177	950	0.4	0.8	3	3.455	5.938	23.48
	**3A**	162	157	797.5	0.3	0.29	1.475	2.078	1.936	10.04
	**5A**	203	190	982.5	0.76	0.86	4.05	6.450	6.843	33.23
	**6A**	227	203	1075	0.4	0.74	2.85	3.864	6.309	25.43
	**7A**	174	163	842.5	0.32	1.42	4.35	2.318	9.690	30.02
	**9A**	168	153	802.5	1.42	0.6	5.05	9.987	3.887	34.69
	**10A**	190	166	890	1.56	0.64	5.5	12.467	4.422	42.22
	**Mean**	193.4	173.6	917.5	0.703	0.745	3.619	5.606	5.45	27.64
	**SEM**	8.494	6.059	35.58	0.179	0.114	0.478	1.335	0.818	3.44
	**SD**	24.02	17.14	100.6	0.508	0.323	1.351	3.776	2.315	9.73
	**N**	8	8	8	8	8	8	8	8	8
**T2DM-P*B.t***	**1P**	240	208	1120	0.7	0.47	2.925	7.045	4.053	27.75
	**2P**	173	150	807.5	1.66	1.89	8.875	12.03	11.88	59.78
	**4P**	190	169	897.5	0.37	0.65	2.55	2.906	4.595	18.75
	**5P**	152	138	725	0.48	1.98	6.15	3.074	11.44	36.28
	**6P**	206	208	1035	1.38	0.64	5.05	11.94	5.548	43.72
	**7P**	216	189	1013	0.36	0.38	1.85	3.197	3.03	15.57
	**9P**	215	222	1093	0.84	1.06	4.75	7.529	9.832	43.4
	**10P**	189	169	895	0.51	0.83	3.35	4.009	5.848	24.64
	**Mean**	197.6	181.6	948.3	0.788	0.988	4.438	6.467	7.028	33.74
	**SEM**	9.752	10.6	49.5	0.172	0.22	0.811	1.358	1.233	5.263
	**SD**	27.58	29.99	140	0.486	0.621	2.294	3.842	3.488	14.89
	**N**	8	8	8	8	8	8	8	8	8

FBG, Fasting Blood Glucose; HOMA-IR, Homeostatic Model Assessment for Insulin Resistance; AUC, Area Under the Curve; NC-PBS, normal control rats gavaged with PBS; NC-*B.t*-EV, normal control rats gavaged with *Bacteroides thetaiotaomicron's* (*B.t*) Extracellular Vesicles; NC-P*B.t*, normal control rats gavages with Pasteurized *B.t*; T2DM-PBS, type 2 diabetes mellitus gavaged with PBS; T2DM-*B.t*-EV, type 2 Diabetes mellitus gavaged with *B.t* EV; T2DM-P*B.t*, type 2 diabetes mellitus gavages with P*B.t*; SEM, standard error of mean; SD, standard deviation; N, number of rats.

EXCLI Journal 2025;24:1743-1766 – ISSN 1611-2156
Received: August 23, 2025, accepted: November 14, 2025, published: December 04, 2025

Supplementary Table 6: Raw data of lipid measures in each group before and after intervention. Raw data is related to Table 1 and Supplementary Figure 2 (g, h, i, j,k, l, m, and n)

Group	rat ID	TG (mg/dL)			TC (mg/dL)			HDL-C (mg/dL)			LDL-C (mg/dL)		
		week 4 (before intervention)	week 9 (after intervention)	AUC (arbitrary unit)	week 4 (before intervention)	week 9 (after intervention)	AUC (arbitrary unit)	week 4 (before intervention)	week 9 (after intervention)	AUC (arbitrary unit)	week 4 (before intervention)	week 9 (after intervention)	AUC (arbitrary unit)
**NC-PBS**	**1S**	41	69	275	67	76	357.5	57.4	45	256	21.3	31.3	131.5
	**2S**	40	46	215	69	54	307.5	52.5	48.9	253.5	26.5	23	123.8
	**3S**	43	72	287.5	47	66	282.5	62	50.1	280.3	15.4	20.4	89.5
	**4S**	62	78	350	68	78	365	43.2	39.1	205.8	27.7	27.3	137.5
	**5S**	64	95	397.5	69	86	387.5	30.4	30.8	153	24.4	36.1	151.3
	**7S**	51	62	282.5	74	60	335	38	44.3	205.8	29	24.9	134.8
	**8S**	75	71	365	84	63	367.5	37.4	46.6	210	33.8	24.9	146.8
	**9S**	72	69	352.5	62	59	302.5	47.7	54.8	256.3	20.2	21.5	104.3
	**Mean**	56.0	70.2	315.6	67.5	67.75	338.1	46.08	44.95	227.6	24.79	26.18	127.4
	**SEM**	4.9	4.8	21.27	3.708	3.913	13.17	3.827	2.594	14.58	2.035	1.864	7.445
	**SD**	14.1	13.8	60.17	10.49	11.07	37.24	10.82	7.336	41.24	5.755	5.272	21.06
	**N**	8	8	8	8	8	8	8	8	8	8	8	8
**NC-*B.t*-EV**	**1A**	32	76	270	56	63	297.5	43.1	39.4	206.3	26.8	25.8	131.5
	**2A**	50	50	250	73	63	340	40.4	42.6	207.5	32.7	24.7	143.5
	**3A**	38	64	255	60	75	337.5	47.7	46.5	235.5	34.5	33.1	169
	**4A**	64	75	347.5	56	72	320	49	38.5	218.8	27.9	31.4	148.3
	**5A**	28	53	202.5	59	69	320	48.8	47.1	239.8	27.3	29.8	142.8
	**6A**	82	53	337.5	66	85	377.5	42.9	37.5	201	33	34.2	168
	**7A**	51	41	230	66	70	340	44	39.6	209	28	35.7	159.3
	**9A**	47	61	270	65	82	367.5	43.5	47	226.3	28.7	38.5	168
	**Mean**	49.0	59.1	270.3	62.6	72.3	337.5	44.93	42.28	218	29.86	31.65	153.8
	**SEM**	6.2	4.3	17.6	2	2.8	9.18	1.119	1.439	5.12	1.069	1.679	5.034
	**SD**	17.6	12.3	49.78	5.9	8	25.98	3.165	4.071	14.48	3.024	4.75	14.24
	**N**	8	8	8	8	8	8	8	8	8	8	8	8
**NC-P*B.t***	**1P**	55	45	250	60	62	305	45.5	47.5	232.5	32.3	31	158.3
	**2P**	60	42	255	79	80	397.5	46.3	51.3	244	29.6	36.1	164.3
	**3P**	63	42	262.5	66	72	345	50.7	50.4	252.8	30.2	33.5	159.3
	**4P**	50	43	232.5	70	71	352.5	49.8	49.7	248.8	33.9	33.5	168.5
	**6P**	58	35	232.5	73	60	332.5	49	43.8	232	31.6	30.6	155.5
	**7P**	59	50	272.5	74	82	390	53	45.5	246.3	36.9	29.9	167
	**8P**	46	33	197.5	63	75	345	56.6	50.3	267.3	22.1	35.8	144.8
	**9P**	29	49	195	75	78	382.5	43.8	52.2	240	34.6	31.7	165.8
	**Mean**	52.5	42.3	237.2	70	72.5	356.3	49.34	48.84	245.5	31.4	32.76	160.4
	**SEM**	3.8	2.1	10.15	2.299	2.841	11.17	1.482	1.044	4.062	1.574	0.83	2.76
	**SD**	11.0	6.0	28.71	6.503	8.036	31.59	4.191	2.952	11.49	4.452	2.347	7.806
	**N**	8	8	8	8	8	8	8	8	8	8	8	8
**T2DM-PBS**	**1S**	131	176	767.5	76	79	387.5	45.8	43.2	222.5	32.9	30.4	158.3
	**2S**	105	147	630	91	91	455	41.5	28.2	174.3	27.1	37.4	161.3
	**3S**	123	150	682.5	97	89	465	34.2	33.3	168.8	34.2	38.8	182.5
	**4S**	111	144	637.5	104	79	457.5	29.5	44.2	184.3	41.2	38.3	198.8
	**5S**	129	164	732.5	78	88	415	46	49	237.5	25.3	34.8	150.3
	**6S**	108	179	717.5	70	99	422.5	33.6	30.5	160.3	39.4	42.2	204
	**8S**	132	173	762.5	93	95	470	44.2	35.7	199.8	37.7	47.8	213.8
	**9S**	119	166	712.5	71	96	417.5	43.5	34.8	195.8	44.6	44	221.5
	**Mean**	119.7	162.3	705.3	85	89.5	436.3	39.79	37.36	192.9	35.3	39.22	186.3

EXCLI Journal 2025;24:1743-1766 – ISSN 1611-2156
Received: August 23, 2025, accepted: November 14, 2025, published: December 04, 2025

	**SEM**	3.7	4.8	18.36	4.543	2.632	10.47	2.261	2.581	9.43	2.383	1.922	9.62
	**SD**	10.7	13.7	51.92	12.85	7.445	29.61	6.395	7.301	26.69	6.741	5.437	27.21
	**N**	8	8	8	8	8	8	8	8	8	8	8	8
**T2DM-*B.t*-EV**	**1A**	123	75	495	77	67	360	39.9	44.3	210.5	35.8	26.4	155.5
	**2A**	96	84	450	74	73	390	40.8	52.8	234	45.1	31.9	192.5
	**3A**	99	87	465	83	68	377.5	45.2	52.3	243.8	43.7	28.9	181.5
	**5A**	102	140	605	70	70	382.5	41.3	50	228.3	45.1	33.5	196.5
	**6A**	114	116	575	82	60	355	45.5	50	238.8	32.7	24.4	142.8
	**7A**	127	122	622.5	88	75	407.5	41.7	52.2	234.8	32	33.6	164
	**9A**	85	94	447.5	96	67	352.5	41.1	43.2	210.8	22.6	32.1	136.8
	**10A**	122	107	572.5	80	60	350	44.2	67.5	279.3	37	29.5	166.3
	**Mean**	108.5	103.1	529.1	81.25	67.5	371.9	42.46	51.54	235	36.75	30.04	167
	**SEM**	5.3	7.7	25.59	2.883	1.918	8.422	0.766	2.619	7.66	2.764	1.188	7.758
	**SD**	15.1	22.0	72.37	8.155	5.425	23.82	2.167	7.407	21.67	7.818	3.359	21.94
	**N**	8	8	8	8	8	8	8	8	8	8	8	8
**T2DM-P*B.t***	**1P**	158	146	760	87	65	380	46.4	39.1	213.8	32.8	29	154.5
	**2P**	96	102	495	78	75	382.5	47.8	33.6	203.5	30.2	36.2	166
	**4P**	101	99	500	75	61	340	50.4	45.4	239.5	23.3	18.2	103.8
	**5P**	114	100	535	91	63	385	30.5	20.8	128.3	35.2	30.3	163.8
	**6P**	95	107	505	96	72	420	45	51.6	241.5	48.4	31.4	199.5
	**7P**	125	150	687.5	84	62	365	44.4	38.1	206.3	45.9	29	187.3
	**9P**	151	138	722.5	91	70	402.5	35.8	40.3	190.3	45.2	28.2	183.5
	**10P**	131	104	587.5	86	74	400	44.8	37.6	206	40.3	31.1	178.5
	**Mean**	121.3	118.2	599.1	86	67.75	384.4	43.14	38.31	203.7	37.67	29.18	167.1
	**SEM**	8.5	7.9	38.43	2.464	1.998	8.67	2.339	3.165	12.46	3.102	1.796	10.38
	**SD**	24.3	22.3	108.7	6.969	5.651	24.52	6.617	8.951	35.23	8.773	5.081	29.36
	**N**	8	8	8	8	8	8	8	8	8	8	8	8

TG, Triglycerides; TC, Total cholesterol; LDL-C, Low-density lipoprotein-cholesterol; HDL-C, High density lipoprotein-cholesterol; AUC, Area Under the Curve; NC-PBS, normal control rats gavaged with PBS; NC-*B.t*-EV, normal control rats gavaged with *Bacteroides thetaiotaomicron*'s (*B.t*) Extracellular Vesicles; NC-P*B.t*, normal control rats gavages with Pasteurized *B.t*; T2DM-PBS, type 2 diabetes mellitus gavaged with PBS; T2DM-*B.t*-EV, type 2 Diabetes mellitus gavaged with *B.t* EV; T2DM-P*B.t*, type 2 diabetes mellitus gavages with P*B.t*; SEM, standard error of mean; SD, standard deviation; N, number of rats.

EXCLI Journal 2025;24:1743-1766 – ISSN 1611-2156
Received: August 23, 2025, accepted: November 14, 2025, published: December 04, 2025

Supplementary Table 7: Raw data of GTT in each group before and after intervention. Raw data is related to Figure 4 (a, b, c, and d)

Group	rat ID	week 4 (before intervention)							week 9 (after intervention)						
		0'	15'	30'	60'	90'	120'	AUC (Arbitrary unit)	0'	15'	30'	60'	90'	120'	AUC (Arbitrary unit)
NC-PBS	1S	105	151	190	146	120	109	15525	104	136	197	110	130	125	16328
	2S	113	147	195	144	144	119	16915	114	155	193	156	110	129	17438
	3S	85	112	197	150	128	98	15215	92	174	166	86	71	103	13290
	4S	92	129	168	142	99	98	13665	100	125	181	134	70	95	14243
	5S	112	176	175	136	140	119	16660	108	142	192	192	133	116	18750
	7S	103	109	166	175	136	118	16425	113	109	193	196	140	99	18390
	8S	94	168	154	154	135	121	16475	98	138	185	126	115	114	15908
	9S	96	151	211	163	111	126	16135	101	152	186	193	199	139	21068
	Mean	100	142.8	182	151.2	126.6	113.5	15877	103.7	141.4	186.6	149.1	121	115	16927
	SEM	3.505	8.63	6.79	4.45	5.49	3.76	374.9	2.67	6.96	3.47	14.81	14.59	5.46	893.4
	SD	9.914	24.4	19.21	12.5	15.54	10.65	1060	7.57	19.71	9.84	41.89	41.28	15.46	2527
	N	8	8	8	8	8	8	8	8	8	8	8	8	8	8
NC-*B.t*-EV	1A	83	130	199	108	121	100	14310	102	109	182	112	99	103	14370
	2A	100	135	195	139	144	136	17140	115	123	175	120	135	126	16185
	3A	84	163	158	160	95	99	14075	105	140	197	158	132	107	17625
	4A	106	161	184	165	143	109	16985	91	160	201	133	96	105	16050
	5A	103	140	189	152	120	102	15305	85	129	162	100	100	97	13673
	6A	97	148	194	128	131	94	15180	80	132	102	75	85	85	10950
	7A	102	118	153	156	119	112	15055	84	135	184	144	104	91	15600
	9A	116	102	158	169	126	107	15440	83	117	145	156	128	120	15960
	Mean	93.13	130.6	168.5	124.8	109.9	104.3	15436	98.88	137.1	178.8	147.1	124.9	107.4	15052
	SEM	4.486	5.487	11.46	10.17	6.696	4.872	393	3.893	7.345	6.766	7.354	5.508	4.582	722.3
	SD	12.69	15.52	32.4	28.77	18.94	13.78	1112	11.01	20.77	19.14	20.8	15.58	12.96	2043
	N	8	8	8	8	8	8	8	8	8	8	8	8	8	8
NC-P*B.t*	1P	105	160	194	163	156	106	17525	100	140	174	124	111	96	15255
	2P	85	95	179	113	113	98	13450	98	121	113	94	100	87	12218
	3P	114	210	210	121	131	130	16985	111	132	183	139	134	107	16725
	4P	111	100	126	151	128	107	14805	108	152	129	139	102	102	14753
	6P	102	168	162	160	101	108	14795	119	128	182	114	120	135	15953
	7P	91	132	192	156	96	89	13805	89	124	176	115	107	101	14663

EXCLI Journal 2025;24:1743-1766 – ISSN 1611-2156
Received: August 23, 2025, accepted: November 14, 2025, published: December 04, 2025

	**8P**	101	125	174	183	111	126	15930	96	148	195	167	140	96	17978
	**9P**	106	126	204	116	134	102	15240	99	99	180	180	153	127	18173
	**Mean**	101.9	139.5	180.1	145.4	121.3	108.3	15317	102.5	130.5	166.5	134	120.9	106.4	15715
	**SEM**	3.435	13.49	9.536	9.053	6.984	4.836	506.1	3.375	5.964	10.28	10.1	6.883	5.8	690.7
	**SD**	9.717	38.16	26.97	25.61	19.75	13.68	1432	9.547	16.87	29.09	28.58	19.47	16.41	1953
	**N**	8	8	8	8	8	8	8	8	8	8	8	8	8	8
**T2DM-PBS**	***1S***	201	276	271	245	195	200	26150	200	290	287	267	258	245	31733
	2S	176	257	317	306	238	201	29480	180	219	308	302	249	183	30840
	3S	227	262	335	318	279	219	32590	235	247	340	285	201	220	30998
	4S	191	210	296	270	245	210	28740	200	250	329	339	266	211	33968
	5S	204	249	314	285	290	236	32480	205	302	339	306	315	258	36195
	6S	198	257	296	317	257	237	31535	202	230	329	329	278	215	33803
	8S	238	292	328	328	317	244	35535	242	276	336	327	239	238	34065
	9S	232	321	319	330	320	283	37050	237	267	332	319	232	228	33203
	**Mean**	208.4	265.5	309.5	299.9	267.6	228.8	31695	212.6	260.1	325	309.3	254.8	224.8	33101
	**SEM**	7.693	11.5	7.327	10.76	14.99	9.765	1262	7.919	10.19	6.486	8.579	11.95	8.185	643.1
	**SD**	21.76	32.52	20.72	30.43	42.41	27.62	3571	22.4	28.83	18.35	24.26	33.8	23.15	1819
	**N**	8	8	8	8	8	8	8	8	8	8	8	8	8	8
**T2DM-*B.t*-EV**	**1A**	220	282	282	277	252	203	29710	180	239	214	196	185	209	24315
	**2A**	203	225	303	289	232	198	28455	177	177	212	216	200	173	23828
	**3A**	162	249	352	290	260	220	30920	157	187	240	200	196	185	24038
	**5A**	203	200	242	262	194	194	25225	190	203	294	271	210	190	28365
	**6A**	227	246	226	248	254	257	29955	203	221	231	234	233	230	27495
	**7A**	174	248	255	277	254	218	29410	163	192	197	182	190	188	22515
	**9A**	168	241	305	296	299	221	32305	153	197	273	240	209	179	26400
	**10A**	190	255	321	289	250	230	30640	166	208	227	200	217	193	24878
	**Mean**	193.4	243.3	285.8	278.5	249.4	217.6	29578	173.6	203	236	217.4	205	193.4	25229
	**SEM**	8.494	8.358	15.09	5.772	10.35	7.169	741.2	6.059	6.987	11.52	10.33	5.51	6.434	707.7
	**SD**	24.02	23.64	42.67	16.33	29.26	20.28	2096	17.14	19.76	32.59	29.22	15.58	18.2	2002
	**N**	8	8	8	8	8	8	8	8	8	8	8	8	8	8
**T2DM-P*B.t***	**1P**	240	241	330	289	309	271	34725	208	237	271	279	258	190	30173
	**2P**	173	220	310	283	195	199	26570	150	200	269	259	245	210	28448
	**4P**	190	283	322	300	284	209	32050	169	227	303	230	240	166	28080
	**5P**	152	179	260	234	259	200	27485	138	205	245	189	190	187	23798
	**6P**	206	208	287	294	248	216	29500	208	258	294	260	226	202	29655
	**7P**	216	266	319	322	298	274	35000	189	233	304	248	237	226	29693
	**9P**	215	323	308	258	256	203	30155	222	224	291	235	219	206	28283

**10P**	189	232	300	312	278	185	30565	169	220	299	250	217	173	27900
**Mean**	197.6	244	304.5	286.5	265.9	219.6	30756	181.6	225.5	284.5	243.8	229	195	28254
**SEM**	9.752	16.09	7.924	10.12	12.57	11.96	1083	10.6	6.478	7.378	9.505	7.387	7.033	704.1
**SD**	27.58	45.51	22.41	28.63	35.57	33.82	3062	29.99	18.32	20.87	26.88	20.89	19.89	1992
**N**	8	8	8	8	8	8	8	8	8	8	8	8	8	8

GTT, Glucose Tolerance Test; AUC, Area Under the Curve; NC-PBS, normal control rats gavaged with PBS; NC-*B.t*-EV, normal control rats gavaged with *Bacteroides thetaiotaomicron*'s (*B.t*) Extracellular Vesicles; NC-P*B.t*, normal control rats gavages with Pasteurized *B.t*; T2DM-PBS, type 2 diabetes mellitus gavaged with PBS; T2DM-*B.t*-EV, type 2 Diabetes mellitus gavaged with *B.t* EV; T2DM-P*B.t*, type 2 diabetes mellitus gavages with P*B.t*; SEM, standard error of mean; SD, standard deviation; N, number of rats.

EXCLI Journal 2025;24:1743-1766 – ISSN 1611-2156
Received: August 23, 2025, accepted: November 14, 2025, published: December 04, 2025

Supplementary Table 8: Raw data of mRNA expression (mean of duplicates) of PI3K, Akt, CB1 and CB2 in the liver in each group. Raw data is related to Figure 5 (a, b, c, and d)

		Fold change ($2^{-\Delta\Delta Ct}$)			
**Group**	**rat ID**	**PI3K**	**Akt**	**CB1**	**CB2**
**NC-PBS**	**1S**	1.0941825	1.048875147	1.058428552	1.061041927
	**2S**	0.8940985	0.950032687	0.929324437	1.061794829
	**3S**	0.9924946	0.939167961	0.993611004	0.950844427
	**4S**	1.2039472	1.085288855	1.034917843	0.96138782
	**5S**	0.9049213	1.040788142	1.081349642	0.928100194
	**7S**	0.9325439	0.931038889	0.928133068	0.955823324
	**8S**	1.0601903	1.008988139	1.021557609	0.9925575
	**9S**	1.0014802	1.018390791	0.975597824	1.115519852
	**Mean**	1.0105	1.0028	1.0029	1.0034
	**SEM**	0.0373	0.0201	0.0200	0.0239
	**SD**	0.1056	0.0569	0.0566	0.0675
	**N**	8	8	8	8
**NC-*B.t*-EV**	**1A**	0.9970425	1.062413178	1.30920423	0.954902752
	**2A**	0.9428314	0.93286738	1.052233841	0.89597673
	**3A**	1.2574326	0.996148975	1.059333196	1.380083194
	**4A**	1.2869463	1.050706993	1.353618145	1.177568467
	**5A**	1.2073413	0.990906242	1.031775612	1.017968789
	**6A**	1.2110051	1.079399565	1.119730056	1.022125022
	**7A**	1.4821425	1.113196908	1.132583164	0.998837065
	**9A**	1.026937	1.013702173	1.359683707	1.199726726
	**Mean**	1.1765	1.0299	1.1773	1.0809
	**SEM**	0.0632	0.0204	0.0496	0.0563
	**SD**	0.1787	0.0576	0.1403	0.1591
	**N**	8	8	8	8
**NC-P*B.t***	**1P**	0.8963681	1.000333	0.966611998	1.162156501
	**2P**	0.9280007	0.958137	1.084807452	1.086352754
	**3P**	0.7891676	0.89417	1.058635274	0.968721664
	**4P**	0.963415	0.911078	1.062811603	1.195238596
	**6P**	0.8736137	0.815725	1.104280487	1.148971299
	**7P**	1.0396775	0.942859	1.274006519	0.952354725
	**8P**	1.0080214	0.986101	1.006463049	1.175215674
	**9P**	1.0039606	0.923364	1.124263877	0.925158412
	**Mean**	0.9378	0.9290	1.0852	1.0768
	**SEM**	0.0294	0.0206	0.0325	0.0393
	**SD**	0.0831	0.0583	0.0918	0.1111

EXCLI Journal 2025;24:1743-1766 – ISSN 1611-2156
Received: August 23, 2025, accepted: November 14, 2025, published: December 04, 2025

	**N**	8	8	8	8
**T2DM-PBS**	**1S**	0.3221797	0.322171999	7.429548032	0.469952435
	**2S**	0.3500577	0.32878988	5.73048905	0.553926866
	**3S**	0.3073377	0.303508214	5.312143819	0.585205205
	**4S**	0.3517578	0.308360328	6.265125224	0.415645564
	**5S**	0.3028835	0.308040882	5.882764788	0.566072893
	**6S**	0.3602718	0.311569253	7.136154849	0.616136855
	**8S**	0.2872009	0.299506714	7.26088719	0.423037522
	**9S**	0.2888293	0.290356092	7.468308246	0.450067799
	**Mean**	0.3213	0.3090	6.5607	0.5100
	**SEM**	0.0104	0.0043	0.3046	0.0279
	**SD**	0.0293	0.0122	0.8615	0.0790
	**N**	8	8	8	8
**T2DM-*B.t*-EV**	**1A**	1.2418218	1.149239137	1.967146101	1.331603902
	**2A**	1.7036086	1.11327263	2.197834562	1.212552293
	**3A**	1.9256097	1.267738854	2.067806392	1.312911111
	**5A**	1.4459283	1.142008467	2.909776295	1.221935612
	**6A**	1.5705642	0.917522978	2.442333168	1.294616826
	**7A**	1.7681963	1.052632033	2.448064916	1.138721616
	**9A**	1.7890621	1.274789875	2.133330854	1.290472527
	**10A**	1.4367054	1.282939675	2.0137113	1.301223551
	**Mean**	1.6102	1.1500	2.2725	1.2630
	**SEM**	0.0801	0.0447	0.1112	0.0232
	**SD**	0.2266	0.1265	0.3146	0.0656
	**N**	8	8	8	8
**T2DM-P*B.t***	**1P**	1.1934956	0.860018555	5.78001277	0.721351008
	**2P**	1.4033305	1.076499838	4.906098593	0.558412315
	**4P**	1.3430747	1.254923493	4.912906678	0.740287346
	**5P**	1.2729396	0.829980969	4.94995325	0.68144172
	**6P**	1.1413853	1.130440902	4.635601917	0.680077897
	**7P**	1.0509217	1.052106551	5.36571569	0.63783567
	**9P**	1.1174537	0.826468145	5.022958423	0.586235445
	**10P**	1.2632202	1.123906643	4.87916084	0.866666467
	**Mean**	1.2232	1.0193	5.0566	0.6840
	**SEM**	0.0421	0.0569	0.1256	0.0342
	**SD**	0.1191	0.1610	0.3552	0.0967
	**N**	8	8	8	8

PI3K, Phosphoinositide 3-kinase; AKT, Protein Kinase B; CB1, Cannabinoid Receptor Type 1; CB2, Cannabinoid Receptor Type 2; NC-PBS, normal control rats gavaged with PBS; NC-*B.t*-EV, normal control rats gavaged with *Bacteroides thetaiotaomicron*'s (*B.t*) Extracellular Vesicles; NC-P*B.t*, normal control rats gavages with Pasteurized *B.t*; T2DM-PBS, type 2 diabetes mellitus gavaged with PBS; T2DM-*B.t*-EV, type 2 Diabetes mellitus gavaged with *B.t* EV; T2DM-P*B.t*, type 2 diabetes mellitus gavages with P*B.t*; SEM, standard error of mean; SD, standard deviation; N, number of rats.

EXCLI Journal 2025;24:1743-1766 – ISSN 1611-2156
Received: August 23, 2025, accepted: November 14, 2025, published: December 04, 2025

Supplementary Table 9: Raw data of mRNA expression (mean of duplicates) of IL1β, IL4, IL6, IL10, CB1 and CB2 in the colon in each group. Raw data is related to Figure 6 (a, b, c, d, e, and f)

		Fold change ($2^{-\Delta\Delta Ct}$)					
**Group**	**rat ID**	**IL1β**	**IL4**	**IL6**	**IL10**	**CB1**	**CB2**
**NC-PBS**	**1S**	1.0500455	0.821813565	0.999450917	1.029495087	1.005835326	0.925651658
	**2S**	1.205987	0.929439694	1.034939943	1.017848207	0.956098737	0.957707981
	**3S**	0.8415593	1.259496917	0.90709162	0.954721315	0.871771363	0.931645508
	**4S**	0.9732983	0.961798452	1.035531692	0.946312078	1.152033131	1.082562968
	**5S**	0.9663778	0.940693572	1.065770535	0.909879894	1.130700151	0.814842137
	**7S**	1.2201959	0.855306901	1.009631972	1.017125534	0.921080752	0.846208271
	**8S**	0.940077	1.073870454	0.93492735	1.107563863	1.015715922	1.279275515
	**9S**	0.8811847	1.268397322	1.028751544	1.032781393	0.990656045	1.286164759
	**Mean**	1.0098	1.0139	1.0020	1.0020	1.0055	1.0155
	**SEM**	0.0495	0.0606	0.0192	0.0220	0.0340	0.0647
	**SD**	0.1401	0.1715	0.0542	0.0623	0.0963	0.1831
	**N**	8	8	8	8	8	8
**NC-*B.t*-EV**	**1A**	1.7060324	1.109980221	0.965479015	0.96871233	1.236359186	0.923597591
	**2A**	1.8135271	0.863909205	1.291632197	0.926592	1.161314129	0.770091997
	**3A**	1.9568793	1.02003105	1.128206053	0.982593873	1.180189232	1.009918509
	**4A**	1.6786015	1.295290484	1.035597381	1.076677289	1.119696026	1.287754616
	**5A**	1.2813077	1.211761625	0.973436114	0.979711114	1.058023221	1.106962972
	**6A**	1.5549283	1.198472004	0.729916455	1.073542884	0.893814136	1.084172851
	**7A**	1.853618	1.329075589	0.968557273	1.010612106	0.946951754	1.211191394
	**9A**	1.4483125	1.410446287	0.942628673	1.058116142	0.996528675	1.780908085
	**Mean**	1.6617	1.1799	1.0044	1.0096	1.0741	1.1468
	**SEM**	0.0792	0.0627	0.0570	0.0194	0.0427	0.1071
	**SD**	0.2239	0.1774	0.1611	0.0549	0.1208	0.3029
	**N**	8	8	8	8	8	8
**NC-P*B.t***	**1P**	1.5383504	1.055967633	0.900181625	1.062903684	0.985925294	1.131995556
	**2P**	1.4752533	1.018164882	1.045147337	1.012212626	0.894461934	0.918581216
	**3P**	1.572616	0.985876096	1.2396029	0.937100634	1.160774113	0.819498596
	**4P**	1.7569935	0.958029763	1.155683585	0.988357117	1.008648906	0.984529496
	**6P**	1.7909962	0.862177836	1.189970758	0.929468249	1.002781497	0.950643366
	**7P**	1.4396673	1.078906508	1.093903889	1.056160391	0.925945809	1.058458306
	**8P**	1.4212301	1.181603588	0.932337522	0.999429992	0.935736883	1.019102115
	**9P**	1.5818809	1.093861874	1.198873322	1.00838253	0.937865941	1.041032573
	**Mean**	1.5721	1.0293	1.0945	0.9993	0.9815	0.9905
	**SEM**	0.0487	0.0342	0.0446	0.0171	0.0293	0.0338
	**SD**	0.1378	0.0968	0.1261	0.0484	0.0828	0.0957
	**N**	8	8	8	8	8	8

EXCLI Journal 2025;24:1743-1766 – ISSN 1611-2156
Received: August 23, 2025, accepted: November 14, 2025, published: December 04, 2025

T2DM-PBS	1S	7.6551253	0.368366964	11.86388213	0.33557473	5.192924568	0.562005828
	2S	6.1293557	0.446429564	10.16716425	0.347725928	4.834990444	0.682279676
	3S	7.5323959	0.439286286	12.82403834	0.341706945	4.718972608	0.492886184
	4S	7.5742665	0.445257386	10.63184506	0.352580001	4.614034257	0.56213937
	5S	6.2149183	0.387894623	11.68031803	0.348026872	4.706990653	0.582375017
	6S	6.4810838	0.460386639	11.46464926	0.369117817	4.942819264	0.55301956
	8S	7.789236	0.369069776	12.54116158	0.335203871	5.428970431	0.467652965
	9S	8.8671552	0.363988683	12.3685037	0.334900323	5.165784129	0.461214649
	**Mean**	7.2804	0.4101	11.6927	0.3456	4.9507	0.5454
	**SEM**	0.3317	0.0146	0.3266	0.0041	0.1012	0.0256
	**SD**	0.9383	0.0414	0.9239	0.0116	0.2862	0.0723
	**N**	8	8	8	8	8	8
T2DM-*B.t*-EV	1A	2.1081206	0.916283833	3.439844433	0.67883745	1.942415321	2.162925271
	2A	2.4424268	0.981568791	3.801564348	0.735693931	1.954937281	2.201215049
	3A	2.3398112	1.114389717	2.695919298	0.759974983	1.839191485	2.996909716
	5A	2.4744589	0.901252501	3.821716414	0.665302062	2.163680524	1.901665615
	6A	3.2501682	0.832142531	4.011103451	0.667729717	2.047887699	1.895038386
	7A	2.1733586	0.979520405	2.774096883	0.730452624	1.871398054	2.039432509
	9A	2.2120392	1.124996349	2.549066884	0.752237889	1.796679722	2.460755167
	10A	2.0149839	1.051553779	3.067559805	0.745804666	1.684669592	2.682462885
	**Mean**	2.3769	0.9877	3.2701	0.7170	1.9126	2.2926
	**SEM**	0.1369	0.0368	0.2027	0.0140	0.0529	0.1386
	**SD**	0.3872	0.1041	0.5734	0.0397	0.1495	0.3921
	**N**	8	8	8	8	8	8
T2DM-P*B.t*	1P	6.5323076	0.596908878	8.289015472	0.560885745	4.003109752	0.844488425
	2P	4.9823234	0.720191632	6.974736863	0.762003798	3.160250569	0.809123023
	4P	4.9598191	1.028162939	7.345205737	0.769719904	2.536270789	0.988587636
	5P	5.4475748	0.869543224	6.888295855	0.64224086	2.957735784	0.832403325
	6P	5.7781725	0.624942717	8.991769067	0.568466934	3.181807454	0.708765219
	7P	5.0940065	0.95505904	7.777019966	0.686815627	3.362380266	0.92092888
	9P	6.4487928	0.673029268	9.15654873	0.468225821	3.711749817	0.644101243
	10P	4.537487	0.939242315	7.174327964	0.701305525	2.8487885	1.107544154
	**Mean**	5.4726	0.8009	7.8246	0.6450	3.2203	0.8570
	**SEM**	0.2567	0.0590	0.3164	0.0374	0.1666	0.0525
	**SD**	0.7261	0.1668	0.8949	0.1058	0.4712	0.1486
	**N**	8	8	8	8	8	8

IL1β, Interleukin-1 beta; IL4, Interleukin-4; IL6, Interleukin-6; IL10, Interleukin-10; CB1, Cannabinoid Receptor Type 1; CB2, Cannabinoid Receptor Type 2; NC-PBS, normal control rats gavaged with PBS; NC-*B.t*-EV, normal control rats gavaged with *Bacteroides thetaiotaomicron*'s (*B.t*) Extracellular Vesicles; NC-P*B.t*, normal control rats gavages with Pasteurized *B.t*; T2DM-PBS, type 2 diabetes mellitus gavaged with PBS; T2DM-*B.t*-EV, type 2 Diabetes mellitus gavaged with *B.t* EV; T2DM-P*B.t*, type 2 diabetes mellitus gavages with P*B.t*; SEM, standard error of mean; SD, standard deviation; N, number of rats.

EXCLI Journal 2025;24:1743-1766 – ISSN 1611-2156
Received: August 23, 2025, accepted: November 14, 2025, published: December 04, 2025

Supplementary Table 10: Raw data of concentration (mean of duplicates) of independent Phylum: *Bacteroidetes*, *Firmicutes*, *Proteobacteria, Actinobacteria* and *Firmicutes/Bacteroidetes* ratio in each group. Raw data is related to Figure 7 (a, and b).

		log 10 copy number per gram				
**Group**	**rat ID**	***Bacteroidetes***	***Firmicutes***	***Proteobacteria***	***Actinobacteria***	***Firmicutes/ Bacteroidetes***
**NC-PBS**	**1S**	7.12393865	7.380404274	5.687319944	4.743734102	1.036000538
	**2S**	7.786331812	7.900477267	4.846004903	5.524984102	1.014659721
	**3S**	7.771069297	7.550720576	5.293843371	4.213709592	0.971644993
	**4S**	7.52381655	7.526389676	5.330084634	5.221675278	1.000341997
	**5S**	7.151411177	7.048895759	5.953952095	5.209420376	0.985665008
	**7S**	7.172778699	7.079309384	5.85299429	5.405498808	0.986968884
	**8S**	7.487186513	7.575051476	5.69767459	4.786626259	1.011735378
	**9S**	7.972534498	7.754491866	5.003913265	4.400596847	0.972650776
	**Mean**	7.49863	7.47697	5.45822	4.93828	0.99746
	**SEM**	0.11569	0.10561	0.14243	0.16857	0.01749
	**SD**	0.32721	0.29871	0.40286	0.47679	0.04948
	**N**	8	8	8	8	8
**NC-*B.t*-EV**	**1A**	7.303800151	7.53550714	7.002360068	4.014567435	1.031724169
	**2A**	7.285551976	7.259645071	7.121438504	5.304395867	0.996444071
	**3A**	7.823873144	7.016450879	7.098140549	6.879150769	0.896800184
	**4A**	7.814749056	6.838592439	4.677741895	6.765792926	0.875087913
	**5A**	6.823264871	7.377612403	5.288666048	4.737606651	1.081243736
	**6A**	7.391999664	8.148937267	5.767568455	6.906724298	1.102399572
	**7A**	7.659639567	8.136233093	5.003913265	4.400596847	1.062221404
	**9A**	7.772169981	7.283238537	4.895189475	4.271920376	0.937092029
	**Mean**	7.48438	7.44953	5.85688	5.41009	0.99788
	**SEM**	0.12361	0.16891	0.37382	0.44242	0.03052
	**SD**	0.34963	0.47776	1.05733	1.25136	0.08631
	**N**	8	8	8	8	8
**NC-P*B.t***	**1P**	6.862802584	6.052099863	5.754625147	5.273758612	0.881870022
	**2P**	6.829347596	6.296250423	5.850405628	5.748636063	0.921940249
	**3P**	7.063532511	7.198251101	5.928065478	4.912239004	1.01907241
	**4P**	6.899298934	7.323717361	5.661433327	5.773145867	1.061516167
	**6P**	7.489323265	7.289807561	5.757213808	5.898758612	0.973359982
	**7P**	7.154773387	7.388145981	6.267180156	6.097900769	1.032617748
	**8P**	6.899298934	6.903235842	5.93065414	5.004150769	1.000570624
	**9P**	7.294676063	7.042266021	6.000548005	4.985768416	0.965398047
	**Mean**	7.06163	6.93672	5.89377	5.46179	0.98204
	**SEM**	0.08368	0.17686	0.06645	0.16627	0.03016

	**SD**	0.23667	0.50023	0.18794	0.47029	0.08531
	**N**	8	8	8	8	8
**T2DM-PBS**	**1S**	6.031142557	8.224532909	7.103317873	7.056846847	1.363677418
	**2S**	6.351655378	7.948438633	6.396613239	6.683072337	1.251396394
	**3S**	6.018932545	8.145969497	6.642536097	7.062974298	1.35339106
	**4S**	5.976197502	8.07862943	6.870338323	6.131601749	1.351800945
	**5S**	6.25397528	8.089852774	7.333708761	6.38895469	1.293553686
	**6S**	7.000827193	7.959661977	7.592574927	6.54520469	1.136960213
	**8S**	6.870636397	8.094342112	7.879916371	7.335645867	1.178106604
	**9S**	7.023273883	8.103320788	7.742717303	7.393856651	1.153781117
	**Mean**	6.44083	8.08059	7.19522	6.82477	1.26033
	**SEM**	0.16048	0.03215	0.18912	0.16169	0.03220
	**SD**	0.45390	0.09093	0.53490	0.45734	0.09107
	**N**	8	8	8	8	8
**T2DM-*B.t*-EV**	**1A**	7.447503973	7.691806376	7.190292005	6.474739004	1.032803259
	**2A**	7.078706205	7.46592115	7.080401895	4.985768416	1.054701372
	**3A**	7.815548311	7.230878415	7.269657085	6.211258612	0.925191442
	**5A**	6.850031069	8.140524325	7.596274911	7.017018416	1.188392322
	**6A**	7.568724354	8.024529209	7.080019918	5.405498808	1.06022215
	**7A**	7.281972993	7.243088427	6.966118805	5.497410573	0.994660161
	**9A**	7.755035948	6.904260588	6.779735165	4.605866455	0.890293821
	**10A**	7.716876576	6.681427866	7.072253933	4.942876259	0.865820232
	**Mean**	7.43930	7.42280	7.12934	5.64255	1.00151
	**SEM**	0.12227	0.18122	0.08434	0.29815	0.03810
	**SD**	0.34582	0.51257	0.23856	0.84329	0.10777
	**N**	8	8	8	8	8
**T2DM-P*B.t***	**1P**	6.934304615	7.306463324	7.489437304	6.238832141	1.053669218
	**2P**	7.137763414	7.495015513	7.153661969	6.637116455	1.050050986
	**4P**	7.293748494	7.147091831	7.257447072	6.1377292	0.979892827
	**5P**	7.324267314	7.64540833	7.092611908	5.57400371	1.043846163
	**6P**	6.964823435	7.735195086	7.361232176	5.319714494	1.110608928
	**7P**	6.937695595	8.080874098	6.830096645	6.110155671	1.164777841
	**9P**	6.598597596	8.004555355	7.31544463	6.735155671	1.213069177
	**10P**	6.913958735	7.346867364	7.278814594	5.92020469	1.062613713
	**Mean**	7.01314	7.59518	7.22234	6.08411	1.08482
	**SEM**	0.08313	0.11927	0.07060	0.17053	0.02662
	**SD**	0.23513	0.33734	0.19969	0.48234	0.07531
	**N**	8	8	8	8	8

NC-PBS, normal control rats gavaged with PBS; NC-*B.t*-EV, normal control rats gavaged with *Bacteroides thetaiotaomicron*'s (*B.t*) Extracellular Vesicles; NC-P*B.t*, normal control rats gavages with Pasteurized *B.t*; T2DM-PBS, type 2 diabetes mellitus gavaged with PBS; T2DM-*B.t*-EV, type 2 Diabetes mellitus gavaged with *B.t* EV; T2DM-P*B.t*, type 2 diabetes mellitus gavages with P*B.t*; SEM, standard error of mean; SD, standard deviation; N, number of rats.

Supplementary Table 11: Raw data of concentration (mean of duplicates) of independent Genus: *Lactobacillus spp., Akkermansia muciniphila, Faecalibacterium prausnitzi, Bacteroides thetaiotaomicron and Clostridium cluster IV* in each group. Raw data is related to Figure 7c.

Group	rat ID	log 10 copy number per gram				
		Lactobacillus spp.	***Akkermansia muciniphila***	***Faecalibacterium Prausnitzi***	***Bacteroides thetaiotaomicron***	***Clostridium Cluster IV***
**NC-PBS**	**1S**	6.189803498	4.1410315	4.531776889	6.145021786	6.657446456
	**2S**	6.114857031	2.574456345	3.958694399	6.064773274	7.297177096
	**3S**	7.112358816	2.490379475	4.005004096	6.116546507	6.11662773
	**4S**	7.173029765	1.639315645	4.485467192	5.997468071	5.767363024
	**5S**	6.971388081	1.375074053	4.297334052	5.330887693	5.231211063
	**7S**	6.685877731	2.732314959	3.709779783	5.46679243	4.983049299
	**8S**	6.40750514	4.026069248	4.138144472	5.623406461	6.478147338
	**9S**	6.955328123	3.966014341	3.508761768	5.790375138	6.720181652
	**Mean**	6.70127	2.86808	4.07937	5.81691	6.15640
	**SEM**	0.14766	0.38156	0.12697	0.11088	0.27901
	**SD**	0.41766	1.07922	0.35913	0.31360	0.78916
	**N**	8	8	8	8	8
**NC-*B.t*-EV**	**1A**	6.40750514	2.219274465	3.122225514	5.865446326	7.629388095
	**2A**	6.296869879	4.062102193	4.123672692	6.749474284	7.027816827
	**3A**	5.418925554	3.794428892	5.26983767	7.280149924	6.668669801
	**4A**	5.716926981	3.708636167	4.520199465	6.82195681	6.958232091
	**5A**	6.105934833	3.578231225	4.068679928	6.726176329	7.039040171
	**6A**	6.46460721	5.390173572	4.459417988	6.908676976	6.895381361
	**7A**	5.882879872	3.327716469	3.449287742	6.674403096	7.285953752
	**9A**	6.277241043	2.898752845	3.417449826	5.927574206	7.555314021
	**Mean**	6.07136	3.62241	4.05385	6.61923	7.13247
	**SEM**	0.13017	0.32559	0.25015	0.17112	0.11731
	**SD**	0.36818	0.92092	0.70752	0.48399	0.33180
	**N**	8	8	8	8	8
**NC-P*B.t***	**1P**	7.276527267	1.795458404	4.589664009	6.498374102	6.583372382
	**2P**	7.317085032	2.068279269	4.685177757	6.577328283	6.661935794
	**3P**	6.911102126	0.348993064	4.123672692	6.143727455	7.339825806
	**4P**	7.051517266	2.505822165	3.834237091	6.172202733	7.276975076
	**6P**	7.00572972	1.042198281	3.191690058	6.353409049	7.02108282
	**7P**	6.557011772	3.132109056	4.106306556	5.85509168	7.456548589
	**8P**	6.312811527	3.140688329	4.36390424	4.914113165	7.429612562
	**9P**	6.050296265	3.053179749	4.178665456	4.866222925	7.088422887
	**Mean**	6.81026	2.13584	4.13416	5.92256	7.10722
	**SEM**	0.16202	0.36572	0.16610	0.23884	0.11873

EXCLI Journal 2025;24:1743-1766 – ISSN 1611-2156
Received: August 23, 2025, accepted: November 14, 2025, published: December 04, 2025

	SD	0.45826	1.03441	0.46980	0.67554	0.33581
	N	8	8	8	8	8
T2DM-PBS	1S	5.813286724	1.298546942	0.434870142	4.766665652	5.681359553
	2S	5.094157531	1.294428892	0.873293788	4.59964997	5.246444712
	3S	5.112001928	0.841443305	-0.377937739	4.07333418	5.143038385
	4S	4.671245325	1.289281328	0.193490832	4.461718935	3.552405782
	5S	5.845406639	0.968416537	0.252604133	4.298379552	3.953865636
	6S	4.767605068	1.72510837	0.631914477	5.018887719	4.428318191
	8S	4.257255318	0.741923744	-0.530647099	4.458089171	4.936225733
	9S	5.424278873	0.424490662	-0.486312124	4.358270659	2.58829386
	Mean	5.12315	1.07295	0.12391	4.50437	4.44124
	SEM	0.19685	0.14434	0.18853	0.10318	0.36314
	SD	0.55678	0.40825	0.53324	0.29183	1.02713
	N	8	8	8	8	8
T2DM-*B.t*-EV	1A	4.777402492	0.815705487	1.922554872	6.033454419	5.542510038
	2A	5.397060612	1.176034931	0.459500684	4.137850023	5.322478775
	3A	5.92514364	1.421402124	1.469352901	6.20439459	6.009826707
	5A	4.908660123	0.678437128	0.070338123	5.236751122	5.459340089
	6A	5.05518027	0.850022577	-0.624243158	4.559095445	5.249486074
	7A	5.360430575	1.097105625	2.53339231	5.386323772	5.745228167
	9A	5.308538023	1.627304664	2.124525315	6.183027069	5.672235466
	10A	5.216962932	1.376789907	1.695953886	6.002929388	6.341335222
	Mean	5.24367	1.13035	1.20642	5.46798	5.66781
	SEM	0.12476	0.11782	0.39246	0.27758	0.12887
	SD	0.35288	0.33325	1.11004	0.78513	0.36450
	N	8	8	8	8	8
T2DM-P*B.t*	1P	5.511716418	1.785290514	-0.028184045	5.780096666	4.958118024
	2P	5.873957674	1.869226838	1.543244527	5.679364065	5.502582634
	4P	5.72406474	2.252729009	1.410239601	5.419901305	5.150495519
	5P	5.711573662	1.931455492	2.794476054	5.951036837	5.593326735
	6P	5.658040472	1.888040152	0.636840586	5.166543552	5.16864434
	7P	5.697298145	1.119411733	0.346200192	5.786201672	5.415468296
	9P	5.586662884	0.9924385	-0.116853995	5.233698619	4.805667933
	10P	5.922137545	1.496899722	1.681175561	5.303906189	5.125087171
	Mean	5.71068	1.66694	1.03339	5.54009	5.21492
	SEM	0.04808	0.15240	0.35362	0.10438	0.09580
	SD	0.13600	0.43104	1.00018	0.29522	0.27097
	N	8	8	8	8	8

NC-PBS, normal control rats gavaged with PBS; NC-*B.t*-EV, normal control rats gavaged with *Bacteroides thetaiotaomicron*'s (*B.t*) Extracellular Vesicles; NC-P*B.t*, normal control rats gavages with Pasteurized *B.t*; T2DM-PBS, type 2 diabetes mellitus gavaged with PBS; T2DM-*B.t*-EV, type 2 Diabetes mellitus gavaged with *B.t* EV; T2DM-P*B.t*, type 2 diabetes mellitus gavages with P*B.t*; SEM, standard error of mean; SD, standard deviation; N, number of rats.

Supplementary Table 12: Raw data of relative phylum abundance (mean of duplicates): *Bacteroidetes*, *Firmicutes*, *Proteobacteria* and *Actinobacteria* in each group. Raw data is related to Figure 8a.

		abundance of 16S taxon-specific (X)*			
**Group**	**rat ID**	***Bacteroidetes***	***Firmicutes***	***Proteobacteria***	***Actinobacteria***
**NC-PBS**	**1S**	0.81894864	0.784555232	0.003675729	0.004451985
	**2S**	1.783326971	1.388686318	0.000258617	0.012846518
	**3S**	2.120790994	0.826541058	0.000995967	0.001143931
	**4S**	1.304745314	0.779903514	0.00108414	0.008513482
	**5S**	1.2141043	0.556538725	0.010220841	0.015878379
	**7S**	1.12209449	0.525516119	0.00700113	0.020839185
	**8S**	1.60770468	1.140448913	0.003671763	0.004718688
	**9S**	2.759849869	1.109522767	0.000418244	0.001465308
	**N**	8	8	8	8
**NC-*B.t*-EV**	**1A**	4.910245541	2.509918761	0.338775625	0.00330488
	**2A**	1.036492561	0.340696856	0.100469703	0.0095244
	**3A**	1.070450009	0.081325989	0.034404453	0.080634428
	**4A**	1.131147454	0.064316998	7.57534E-05	0.069141975
	**5A**	0.608536035	0.601371088	0.001331018	0.004415135
	**6A**	0.455003056	0.566045078	0.001124006	0.083707425
	**7A**	3.828259939	2.772114416	0.000797835	0.002795198
	**9A**	5.172786219	0.689069407	0.000656215	0.002347522
	**N**	8	8	8	8
**NC-P*B.t***	**1P**	0.151837118	0.012642389	0.001012989	0.002980016
	**2P**	0.196919584	0.027488644	0.001791041	0.010656385
	**3P**	0.564353903	0.265499858	0.003975021	0.003642506
	**4P**	0.244502764	0.19952772	0.001197386	0.012132493
	**6P**	0.78023192	0.190366501	0.001554101	0.015843446
	**7P**	2.247832827	1.259003582	0.031579691	0.1300612
	**8P**	0.594222163	0.222700642	0.005794023	0.006338175
	**9P**	1.294753633	0.291469259	0.00701063	0.006182089
	**N**	8	8	8	8
**T2DM-PBS**	**1S**	0.028485941	0.733998598	0.039545287	0.130474887
	**2S**	0.041624849	0.266776465	0.005101302	0.048605466
	**3S**	0.021794383	0.461604987	0.009531686	0.103469021
	**4S**	0.054132565	1.031564267	0.046049543	0.043367911
	**5S**	0.064459857	0.740932544	0.104868508	0.050496462
	**6S**	0.110501524	0.567032937	0.223936263	0.076001231
	**8S**	0.031849313	0.335335821	0.189532979	0.14977532
	**9S**	0.045824041	0.331233252	0.128534887	0.162080973
	**N**	8	8	8	8

EXCLI Journal 2025;24:1743-1766 – ISSN 1611-2156
Received: August 23, 2025, accepted: November 14, 2025, published: December 04, 2025

**T2DM-*B.t*-EV**	**1A**	0.503172775	0.361945904	0.097461715	0.046588946
	**2A**	0.535239179	0.452279805	0.152667827	0.004720435
	**3A**	1.056350463	0.166208441	0.137134187	0.032588732
	**5A**	0.131014335	0.642562864	0.165941098	0.09721603
	**6A**	1.554606443	1.86768907	0.110906378	0.014307056
	**7A**	1.002687191	0.38510551	0.085191085	0.017673439
	**9A**	1.881896315	0.23830187	0.065675408	0.003692634
	**10A**	0.959824256	0.084982815	0.078256775	0.004084026
	**N**	8	8	8	8
**T2DM-P*B.t***	**1P**	0.309085051	0.092033065	0.266341849	0.041518799
	**2P**	0.277791372	0.09835002	0.081096601	0.058068272
	**4P**	0.342155319	0.034912153	0.095370673	0.020090954
	**5P**	0.760991955	0.296555499	0.140675449	0.013729753
	**6P**	0.479452288	0.448845782	0.296662627	0.009724377
	**7P**	0.275183321	0.709873495	0.056396621	0.028412409
	**9P**	0.149861259	0.563406584	0.158783585	0.097374323
	**10P**	0.244531806	0.084456336	0.13824272	0.017998657
	**N**	8	8	8	8

Eff. Univ, efficiency of the universal primers; Eff. Spec, efficiency of the taxon-specific primers; NC-PBS, normal control rats gavaged with PBS; NC-*B.t*-EV, normal control rats gavaged with *Bacteroides thetaiotaomicron's* (*B.t*) Extracellular Vesicles; NC-P*B.t*, normal control rats gavages with Pasteurized *B.t*; T2DM-PBS, type 2 diabetes mellitus gavaged with PBS; T2DM-*B.t*-EV, type 2 Diabetes mellitus gavaged with *B.t* EV; T2DM-P*B.t*, type 2 diabetes mellitus gavages with P*B.t*; SEM, standard error of mean; SD, standard deviation; N, number of rats.
*X represents the percentage of 16S taxon-specific abundance existing in a sample:

$$x = \frac{(Eff.Univ)^{\text{CT univ}}}{(Eff.Sppec)^{CT\ spec}} x100$$

In Figure 8a after calculating X for each sample, group means were obtained, and the total abundance of targeted bacteria was determined. The relative abundance of each bacterium was then expressed as its proportion of the total bacterial load (%).

EXCLI Journal 2025;24:1743-1766 – ISSN 1611-2156
Received: August 23, 2025, accepted: November 14, 2025, published: December 04, 2025

Supplementary Table 13: Raw data of genus abundance (mean of duplicates): *Lactobacillus spp., Akkermansia muciniphila, Faecalibacterium prausnitzi, Bacteroides thetaiotaomicron and Clostridium cluster IV* in each group. Raw data is related to Figure 8b.

		abundance of 16S taxon-specific (X)*				
**Group**	**rat ID**	***Lactobacillus spp.***	***Akkermansia Muciniphila***	***Faecalibacterium Prausnitzi***	***Bacteroides thetai-otaomicron***	***Clostridium Cluster IV***
**NC-PBS**	**1S**	0.012002728	0.016893758	0.012736618	0.070042567	0.112313908
	**2S**	0.005645346	0.000656149	0.002260917	0.035241253	0.357846681
	**3S**	0.198411477	0.00069354	0.003057811	0.048630125	0.122319692
	**4S**	0.241442503	0.000154782	0.008484105	0.036677658	0.062452076
	**5S**	0.234133529	0.00018616	0.010840895	0.01510543	0.043049531
	**7S**	0.079440472	0.001782449	0.002733627	0.018318091	0.02381211
	**8S**	0.024292255	0.013434805	0.005335561	0.020499996	0.319269588
	**9S**	0.103120735	0.008124066	0.017615325	0.020229833	0.339911736
	**N**	8	8	8	8	8
**NC-*B.t*-EV**	**1A**	0.07963668	0.001854931	0.001988268	0.11734072	4.466947816
	**2A**	0.012002248	0.010259978	0.003708603	0.196494144	0.204904681
	**3A**	0.000227328	0.002332507	0.015670861	0.242386394	0.029313014
	**4A**	0.000666511	0.002158476	0.003387696	0.002888007	0.066864209
	**5A**	0.009086999	0.006323615	0.004745601	0.268071322	0.303673085
	**6A**	0.00753646	0.037583661	0.002716869	0.101233459	0.051878908
	**7A**	0.005327234	0.005061837	0.001565302	0.295487887	0.715939126
	**9A**	0.021815036	0.002592061	0.001588249	0.057490515	1.564971362
	**N**	8	8	8	8	8
**NC-P*B.t***	**1P**	0.107885217	6.41927E-05	0.003344679	0.03666231	0.021498253
	**2P**	0.166341463	0.000143313	0.005678538	0.060851904	0.036482416
	**3P**	0.136180442	1.28032E-05	0.003106335	0.04079588	0.385827125
	**4P**	0.106948963	0.000334547	0.000996099	0.025953307	0.1952657
	**6P**	0.099288658	2.61224E-05	0.000255777	0.040017266	0.102079389
	**7P**	0.226435086	0.005616989	0.009987277	0.070034234	1.743187
	**8P**	0.060776811	0.002474336	0.007521792	0.003481107	0.705335971
	**9P**	0.036693757	0.00214767	0.005119916	0.003154685	0.294321197
	**N**	8	8	8	8	8
**T2DM-PBS**	**1S**	0.000972122	3.32956E-05	0.000162351	0.003025764	0.022240935
	**2S**	6.79867E-05	2.59971E-05	0.000221605	0.003833629	0.007601514
	**3S**	7.19106E-05	1.17044E-05	4.57607E-05	0.001608899	0.006210654
	**4S**	4.40186E-05	6.92282E-05	0.000253264	0.008213228	0.000795123
	**5S**	0.001593193	2.74573E-05	0.000189895	0.004371947	0.001194473
	**6S**	4.66679E-05	0.000113932	0.000336962	0.015719782	0.003265178
	**8S**	3.4003E-06	8.2504E-06	3.16944E-05	0.002539985	0.003506917
	**9S**	0.00016627	4.55574E-06	3.22811E-05	0.00207679	3.75647E-05

EXCLI Journal 2025;24:1743-1766 – ISSN 1611-2156
Received: August 23, 2025, accepted: November 14, 2025, published: December 04, 2025

	**N**	8	8	8	8	8
**T2DM-*B.t*-EV**	**1A**	0.001152171	1.63282E-05	0.001205374	0.041050848	0.025875871
	**2A**	0.007750959	6.10529E-05	0.000380625	0.002014372	0.02541504
	**3A**	0.013050176	5.59254E-05	0.000807498	0.067894485	0.056518001
	**5A**	0.001052681	9.05889E-06	8.28231E-05	0.006113579	0.011726914
	**6A**	0.005181168	4.51502E-05	0.000127571	0.006005411	0.028940172
	**7A**	0.009709261	7.15716E-05	0.00695513	0.03105062	0.07691637
	**9A**	0.010887222	0.000225142	0.005164901	0.182727087	0.083050163
	**10A**	0.005126693	8.1826E-05	0.001698824	0.07248063	0.168774573
	**N**	8	8	8	8	8
**T2DM-P*B.t***	**1P**	0.000575333	2.48399E-05	0.000148074	0.035746509	0.014460729
	**2P**	0.001228048	1.86559E-05	0.000673961	0.018522653	0.02186414
	**4P**	0.00069629	3.88709E-05	0.000535338	0.010478371	0.011664481
	**5P**	0.001408091	4.20454E-05	0.006437868	0.062349733	0.050088441
	**6P**	0.001384748	4.52382E-05	0.000502687	0.015867106	0.029825775
	**7P**	0.000950866	3.51494E-05	0.000209863	0.032017905	0.026673031
	**9P**	0.000643777	2.7641E-05	0.000115169	0.01069375	0.00984409
	**10P**	0.001876551	6.30894E-05	0.001041598	0.011561592	0.015496931
	**N**	8	8	8	8	8

Eff. Univ, efficiency of the universal primers; Eff. Spec, efficiency of the taxon-specific primers; NC-PBS, normal control rats gavaged with PBS; NC-*B.t*-EV, normal control rats gavaged with *Bacteroides thetaiotaomicron*'s (*B.t*) Extracellular Vesicles; NC-P*B.t*, normal control rats gavages with Pasteurized *B.t*; T2DM-PBS, type 2 diabetes mellitus gavaged with PBS; T2DM-*B.t*-EV, type 2 Diabetes mellitus gavaged with *B.t* EV; T2DM-P*B.t*, type 2 diabetes mellitus gavages with P*B.t*; SEM, standard error of mean; SD, standard deviation; N, number of rats.

*X represents the percentage of 16S taxon-specific abundance existing in a sample:

$$x = \frac{(Eff.Univ)^{\text{CT univ}}}{(Eff.Sppec)^{CT\ spec}} x100$$

In Figure 8b after calculating X for each sample, group means were obtained, and the total abundance of targeted bacteria was determined. The relative abundance of each bacterium was then expressed as its proportion of the total bacterial load (%).
